# Inhibition of GluR Current in Microvilli of Sensory Neurons via Na^+^-Microdomain Coupling Among GluR, HCN Channel, and Na^+^/K^+^ Pump

**DOI:** 10.3389/fncel.2018.00113

**Published:** 2018-04-24

**Authors:** Yasuhiro Kawasaki, Mitsuru Saito, Jonghwa Won, Jin Young Bae, Hajime Sato, Hiroki Toyoda, Eriko Kuramoto, Mikihiko Kogo, Takuma Tanaka, Takeshi Kaneko, Seog Bae Oh, Yong Chul Bae, Youngnam Kang

**Affiliations:** ^1^Department of Neuroscience and Oral Physiology, Graduate School of Dentistry, Osaka University, Osaka, Japan; ^2^Department of Brain and Cognitive Sciences, College of Natural Sciences, Dental Research Institute and Department of Neurobiology and Physiology, School of Dentistry, Seoul National University, Seoul, South Korea; ^3^Department of Oral Anatomy, School of Dentistry, Kyungpook National University, Daegu, South Korea; ^4^Department of Morphological Brain Science, Graduate School of Medicine, Kyoto University, Kyoto, Japan; ^5^Department of Computational Intelligence and Systems Science, Interdisciplinary Graduate School of Science and Engineering, Tokyo Institute of Technology, Yokohama, Japan

**Keywords:** glutamate receptor, hyperpolarization-activated cyclic nucleotide-gated cation channel, Na^+^/K^+^ pump, primary sensory neuron, mesencephalic trigeminal nucleus, microvilli

## Abstract

Glutamatergic dendritic EPSPs evoked in cortical pyramidal neurons are depressed by activation of hyperpolarization-activated cyclic nucleotide-gated (HCN) channels expressed in dendritic spines. This depression has been attributed to shunting effects of HCN current (*I*_h_) on input resistance or *I*_h_ deactivation. Primary sensory neurons in the rat mesencephalic trigeminal nucleus (MTN) have the somata covered by spine-like microvilli that express HCN channels. In rat MTN neurons, we demonstrated that *I*_h_ enhancement apparently diminished the glutamate receptor (GluR) current (*I*_GluR_) evoked by puff application of glutamate/AMPA and enhanced a transient outward current following *I*_GluR_ (OT-*I*_GluR_). This suggests that some outward current opposes inward *I*_GluR_. The *I*_GluR_ inhibition displayed a U-shaped voltage-dependence with a minimal inhibition around the resting membrane potential, suggesting that simple shunting effects or deactivation of *I*_h_ cannot explain the U-shaped voltage-dependence. Confocal imaging of Na^+^ revealed that GluR activation caused an accumulation of Na^+^ in the microvilli, which can cause a negative shift of the reversal potential for *I*_h_ (*E*_h_). Taken together, it was suggested that *I*_GluR_ evoked in MTN neurons is opposed by a transient decrease or increase in standing inward or outward *I*_h_, respectively, both of which can be caused by negative shifts of *E*_h_, as consistent with the U-shaped voltage-dependence of the *I*_GluR_ inhibition and the OT-*I*_GluR_ generation. An electron-microscopic immunohistochemical study revealed the colocalization of HCN channels and glutamatergic synapses in microvilli of MTN neurons, which would provide a morphological basis for the functional interaction between HCN and GluR channels. Mathematical modeling eliminated the possibilities of the involvements of *I*_h_ deactivation and/or shunting effect and supported the negative shift of *E*_h_ which causes the U-shaped voltage-dependent inhibition of *I*_GluR_.

## Introduction

To date, many studies have reported that, not only in cortical pyramidal cells but also in various other neurons, the activation of hyperpolarization-activated cyclic nucleotide-gated (HCN) cation channels can decrease the amplitude and/or duration of EPSPs or depolarizations evoked by current pulses (Magee, 1998, 1999; Yamada et al., 2005; Carr et al., 2007; Ying et al., 2007; Harnett et al., 2015). Indeed, the blockade of HCN channels increased the amplitudes of EPSPs or depolarizations in these studies. Such modulation of EPSPs by HCN channels is crucially involved in a variety of brain functions, such as working memory (Wang et al., 2007), sleep/wakefulness (Postea and Biel, 2011), epilepsy (DiFrancesco et al., 2011), autism (Yi et al., 2016), and neuropathic pain (Harnett et al., 2015). Originally, the deactivation of HCN channels-mediated current (*I*_h_) by EPSPs was considered to be responsible for the diminution of EPSPs and the generation of hyperpolarization following EPSPs, based on their sensitivity to *I*_h_ blockers (Magee, 1998, 1999). However, it is not clear whether the abolishment of the afterhyperpolarization by *I*_h_ blockers is a direct consequence of the abolishment of *I*_h_ deactivation or secondary to the *I*_h_ blocker-induced negative shift of the baseline membrane potential that consequently attenuates K^+^ channel-mediated afterhyperpolarization.

Since HCN channels were found to be expressed in the apical dendrites, especially on the shafts of dendritic spines, of cortical pyramidal cells (Lörincz et al., 2002; Notomi and Shigemoto, 2004), the inhibition of EPSPs by the activity of HCN channels has been considered to be due to the shunting effects of HCN channels on the input impedance of the spine that receives excitatory synaptic inputs (Carr et al., 2007; Tsay et al., 2007; Wang et al., 2007; Harnett et al., 2015). However, it is also not clear whether the peak level of EPSPs is lowered by a shunting effect of increases in the HCN channel conductance (*G*_h_) although it certainly decreases the amplitudes of EPSPs, because *G*_h_ increases would depolarize the baseline membrane potential toward the reversal potential for *I*_h_ (*E*_h_), near −40 mV. Indeed, the peak level of the EPSP observed following the blockade of *I*_h_ with ZD7288 was not higher than that of the control due to the hyperpolarization of the baseline potential, while this was not necessarily the case for that of summated EPSPs (Carr et al., 2007), indicating that the shunting effect is not always effective. Subsequently, it has been proposed in a mathematical simulation study that, in CA1 hippocampal pyramidal neurons, HCN-mediated depolarization can secondarily activate M-type K^+^ channels or some other K^+^ channels, which can produce a real shunting conductance with a more negative reversal potential (George et al., 2009; Migliore and Migliore, 2012). Thus, it remains unclear and controversial how *I*_h_ diminishes EPSPs despite its crucial involvement in various brain functions.

Among all the primary sensory neurons, those innervating muscle spindles in the jaw-closing muscles are uniquely and exceptionally located in the brain stem as the mesencephalic trigeminal nucleus (MTN), thereby receiving peptidergic, catecholaminergic, serotonergic, and nitrergic perineuronal arborizations in a basket-like manner in addition to glutamatergic input and expressing various receptors (Lazarov, 2002) including glutamate receptors (GluRs; Mineff et al., 1998; Turman et al., 2000) inducing DNQX/AP5-sensitive glutamatergic EPSPs (Verdier et al., 2004). MTN neurons have no dendrites but express numerous spine-like microvilli directly protruding from the somata (Liem et al., 1991), in which HCN channels are expressed (Kang et al., 2004). In the present study, we explored whether and how GluR-mediated currents (*I*_GluR_) are modified by concurrent activation of *I*_h_, under voltage-clamp conditions in MTN neurons by taking advantages of their characteristic morphological structure of the round shaped soma covered by short spine-like microvilli, where space-clamp errors would not occur.

## Materials and methods

### Ethical approval

The experimental protocols were approved either by the Animal Ethics Committees of the Osaka University Graduate School of Dentistry for the Care and Use of Laboratory Animals or by Kyungpook National University Intramural Animal Care and Use Committee, and all experiments were performed in accordance with the relevant guidelines.

### Slice preparation

Wistar and Sprague-Dawley (SD) rats of both sexes at postnatal day (PND) 13–18 were used for the experiments shown in the results and the Supplemental Material, respectively. *I*_h_ has been reported to be matured at PND 10–12 in MTN neurons (Tanaka et al., 2003), and various synaptic inputs including glutamatergic one are developmentally mature by PND 11 (Paik et al., 2012). Therefore, rats at PND 13–18 can be used in place of adult preparations to investigate *I*_h_ and *I*_GluR_ in MTN neurons. The rats were anesthetized with isoflurane, and the brains were quickly removed from the skull and immersed in ice-cold modified artificial cerebrospinal fluid (ACSF) containing the following (in mM): 210 sucrose, 1.8 KCl, 1.2 KH_2_PO_4_, 26 NaHCO_3_, 0.5 CaCl_2_, 2.5 MgCl_2_, and 50 d-glucose. With a microslicer (Super ZERO-1, Dosaka EM, Kyoto, Japan), coronal sections of 250 μm thickness including the MTN were cut and incubated at room temperature (20–24°C) for 30 min in 50% modified ACSF and 50% normal ACSF (N-ACSF, pH 7.3) containing the following (in mM): 124 NaCl, 1.8 KCl, 1.2 KH_2_PO_4_, 26 NaHCO_3_, 2.0 CaCl_2_, 1.0 MgCl_2_, and 10 d-glucose. The slices were then placed in N-ACSF at room temperature. N-ACSF was continuously gassed with a mixture of 95% O_2_-5% CO_2_.

### Whole-cell patch-clamp recordings

Using an Axopatch 1D (MDS Analytical Technologies, Sunnyvale, CA), whole-cell voltage-clamp or current-clamp recordings were made from MTN neurons that were viewed under Nomarski optics (BX50WI-DIC, Olympus, Tokyo, Japan). The recording chamber, with a volume of 1.0 ml, was continuously perfused with the extracellular solution (N-ACSF) at a flow rate of 1.0–1.5 ml/min. The internal solution of the patch pipettes had the following ionic composition (in mM): 123 K-gluconate, 18 KCl, 10 NaCl, 2 MgCl_2_, 2 ATP-Na_2_, 0.3 GTP-Na_3_, 10 HEPES, and 0.2 EGTA; pH 7.3 adjusted with KOH (Tanaka et al., 2003; Kang et al., 2004). The membrane potential values given in the text were corrected for the junction potential (10 mV) between the internal solution for the whole-cell recording (negative) and the standard extracellular solution. The pipette resistances were 4–6 MΩ. The series resistance was <10 MΩ. All recordings were made at room temperature. Series resistance was compensated by ~70% when the *I-V* relationships were measured while it was not performed when current responses were recorded at a fixed holding potential. This is mainly because the activation time constants of puff-induced *I*_GluR_ (>50 ms) or *I*_h_ (>100 ms) in MTN neurons (Tanaka et al., 2003) were much slower than the time constant of the capacitative current in MTN neurons (<5 ms). Records of currents and voltages were low-pass filtered at 5 kHz (3-pole Bessel filter), digitized at a sampling rate of 40 kHz (Digidata 1322A, MDS Analytical Technologies) and stored on a computer hard disk.

### Drug application

Using a pressure-pulsed microinjector (Picopump PV820, World Precision Instruments, Sarasota, FL), 50–200 μM glutamate or α-amino-3-hydroxy-5-methylisoxazole-4-propionic acid hydrate (AMPA; Sigma-Aldrich, St. Louis, MO) was puff-applied for 50 or 20–500 ms, respectively, through a glass pipette, the tip of which was placed 10–20 μm apart from the soma. CsCl (an *I*_h_ blocker), ZD7288 (an *I*_h_ blocker), 8-bromoadenosine 3′,5′-cyclic monophosphate sodium salt (8-Br-cAMP; a membrane-permeable cAMP analog), 8-bromoguanosine 3′,5′-cyclic monophosphate sodium salt (8-Br-cGMP; a membrane-permeable cGMP analog), and ouabain octahydrate (a Na^+^/K^+^ pump inhibitor) were bath-applied at 5 mM, 10, 500, 200, and 50–100 μM, respectively. These chemicals were purchased from Sigma-Aldrich. ZD7288 is also known to block Na_v_1.4 (Wu et al., 2012) and T-type Ca^2+^ currents (Sánchez-Alonso et al., 2008). Because it has been reported that HCN channels localized in the presynaptic terminal are involved in the modulation of glutamate release (Huang et al., 2011; Huang and Trussell, 2014), we employed puff application of glutamate or AMPA to isolate the effects of postsynaptic HCN channels on the postsynaptic GluR, instead of examining the responses caused by activation of presynaptic input pathways. On the other hand, puff application of AMPA or glutamate may cause strong desensitization because the puff duration is much larger than the duration of synaptic transmission. Given the desensitization of AMPA currents depending on the concentration of AMPA or glutamate and the duration of puff application, we may have underestimated the effects of HCN channel activity on AMPA currents. However, such desensitization does not preclude our conclusion regarding whether HCN activity effectively inhibits GluR currents if it does despite the desensitization.

### Fluorescence imaging of Na^+^ transient with sodium green tetraacetate

Sodium Green tetraacetate and Pluronic F-127 were purchased from Thermo Fisher Scientific (Waltham, MA). The stock solution was prepared by dissolving 5 mM Sodium Green tetraacetate in DMSO and mixing it with an equal volume of 25% w/v Pluronic F-127 (Friedman and Haddad, 1994; Amorino and Fox, 1995). Slice preparations including the MTN neurons were incubated for 60 min in oxygenated ACSF containing 10 μM Sodium Green tetraacetate and then washed in the ACSF for 30 min before optical recording of the glutamate responses. Sodium Green-loaded slices were submerged in a chamber placed on the stage of a confocal microscope (LSM510; Carl Zeiss Microscopy GmbH, Jena, Germany). The sodium imaging was performed with an excitation of Sodium Green at 488 nm and its emission at >510 nm. We have not attempted the calibration of Sodium Green because it would largely underestimate the rapid and large changes in Na^+^ concentration in beneath the membrane in microvilli caused by activation of GluR due to the possible slow binding rate constant as a consequence of a large dissociation constant (6–21 mM) of Sodium Green.

### Electron-microscopic immunohistochemistry

Three male SD rats weighing 300–320 g (8 weeks old) were used for this study. For tissue fixation, the rats were deeply anesthetized with sodium pentobarbital (80 mg/kg, i.p.) and perfused transcardially with 100 ml of heparinized normal saline followed by 500 ml of a freshly prepared mixture of 4% paraformaldehyde and 0.01% glutaraldehyde in 0.1 M phosphate buffer (PB; pH 7.4). The brainstem was removed and post-fixed in the same fixative for 2 h at 4°C. Sections were cut transversely on a vibratome at 60 μm and cryoprotected in 30% sucrose in PB overnight at 4°C. The sections were frozen on dry ice for 20 min and then thawed in 0.01 M phosphate-buffered saline (pH 7.2) to enhance penetration. The slices were pretreated with 1% sodium borohydride for 30 min to quench the glutaraldehyde and then blocked with 10% normal donkey serum (Jackson ImmunoResearch, West Grove, PA) for 30 min to mask the secondary antibody binding sites. For single immunostaining for vesicular glutamate transporter 2 (VGLUT2), the sections of brainstem were incubated overnight in mouse anti-VGLUT2 (1:1,000; MAB5504, Merck Millipore, Billerica, MA) antibody. After rinsing in phosphate-buffered saline, the sections were incubated with 1 nm gold-conjugated donkey anti-rabbit (1:50; EMS, Hatfield, PA) antibody for 2–3 h. The sections were post-fixed with 1% glutaraldehyde in PB for 10 min, rinsed in PB several times, incubated for 4 min with HQ silver enhancement solution (Nanoprobes, Yaphank, NY) and rinsed in 0.1 M sodium acetate and PB. To control for the specificity of the antibody, the sections were processed as described above, except that the primary or secondary antibodies were omitted. Omission of the primary or secondary antibodies eliminated specific staining. Pre-adsorption with blocking peptides for VGLUT2 (15 mg/ml; #135-40P, Synaptic Systems) also completely abolished the respective staining. For immunostaining for HCN or glutamate was described in our previous studies (Cho et al., 2015; Park et al., 2016).

Sections were osmicated (in 0.5% osmium tetroxide in PB) for 30 min, dehydrated in graded alcohols, flat-embedded in Durcupan ACM (Fluka, Buchs, Switzerland) between strips of Aclar plastic film (EMS), and cured for 48 h at 60°C. Chips containing prominent staining for VGLUT2 in the brainstem containing MTN were cut out of the wafers and glued onto blank resin blocks with cyanoacrylate. Serially cut thin sections were collected on Formvar-coated single-slot nickel grids and stained with uranyl acetate and lead citrate. The grids were examined on a Hitachi H-7500 electron microscope (Hitachi, Tokyo, Japan) at 80 kV accelerating voltage. Images were captured with Digital Montage software driving a MultiScan cooled CCD camera (ES1000W; Gatan, Pleasanton, CA) attached to the microscope and saved as TIFF files.

### Statistical analysis

Normal distribution of data and homogeneity of variance were checked by Kolmogorov-Smirnov Lilliefors test and Levene's test, respectively (*P* > 0.05). Numerical data are expressed as the mean ± the SD (parametric) or the median with the interquartile range (IQR; non-parametric). Statistical significance of mean difference was assessed using paired Student's *t*-tests (^*^), while that of median difference was assessed using Wilcoxon signed-rank test (†). The Pearson correlation coefficient (#) was calculated to assess the strength of a linear association between the two variables. *P* < 0.05 was considered statistically significant.

### Mathematical modeling

*I*_GluR_ can be expressed as follows:

$$\begin{array}{l} I_{\text{GluR}}=N_{0}P\left(t\right)\left\{i_{\text{Na}}\left(t\right)+i_{\text{K}}\left(t\right)\right\},\\ P\left(t\right)={\left(\frac{\tau_{1}+\tau_{2}}{\tau_{1}}\right)}^{\frac{\tau_{1}}{\tau_{2}}}\frac{\tau_{1}+\tau_{2}}{\tau_{2}}\;\left\{1-\exp\left(-t/\tau_{1}\right)\right\}\;exp\left(-t/\tau_{2}\right), \end{array}$$

where *N*_0_ is the maximum number of activated GluR channels, *P*(*t*) represents the time course of open probability change of the GluR channels (0 ≤ *P*(*t*) ≤ 1), τ_1_ and τ_2_ are the time constants for the rising and decay phases of open probability, respectively, and a single GluR current is expressed as the sum of *i*_Na_(*t*) and *i*_K_(*t*) because the GluR channel is equally permeable to Na^+^ and K^+^. *i*_Na_(*t*) and *i*_K_(*t*) should follow the Goldman-Hodgkin-Katz equation and can be expressed as follows:

$$\begin{array}{l} i_{\text{X}}\left(t\right)=\;k\frac{V\left(t\right)F^{2}}{RT}\frac{X_{i}\left(t\right)-X_{\text{o}}\left(t\right)\exp\left\{-V\left(t\right)F/RT\right\}}{1-\exp\left\{-V\left(t\right)F/RT\right\}}\;\left(\text{X}:\text{ N}{\text{a}}^{+}\text{ or }{\text{K}}^{+}\right), \end{array}$$

where *V*(*t*), *F, R*, and *T* are the membrane potential, Faraday constant, gas constant and absolute temperature, respectively, and the coefficient *k* (= 1.22 × 10^−17^) was introduced to yield a single GluR current of 0.5 pA at −70 mV (Swanson et al., 1997). Provided that an MTN neuron is composed of the soma and microvilli compartments, the following first order differential equations can be formulated:

$$\begin{array}{l} \frac{V_{S}\left(t\right)-V_{V}\left(t\right)}{R_{i}}=C_{V}\frac{dV_{V}\left(t\right)}{dt}+N_{0}P\left(t\right)\left\{i_{Na}\left(t\right)+i_{K}\left(t\right)\right\}\\ +\frac{V_{V}\left(t\right)-E_{K}}{R_{V}}+G_{h-V}\left(t\right)\left\{V_{V}\left(t\right)-E_{h}\left(t\right)\right\},\\ \frac{V_{V}\left(t\right)-V_{S}\left(t\right)}{R_{i}}=C_{S}\frac{dV_{S}\left(t\right)}{dt}+\;\frac{V_{S}\left(t\right)-E_{K}}{R_{S}}\\ +\;G_{h-S}\left(t\right)\left\{V_{S}\left(t\right)-E_{h}\left(t\right)\right\},\\ ssG_{h}\left(V\left(t\right)\right)\;=\;G_{hMax}/\left[1+\exp\left\{\frac{V\left(t\right)-V_{half}}{S_{f}}\right\}\right]\;,\\ \frac{dG_{h}\left(t\right)}{dt}\;=\;\frac{ssG_{h}\left(V\left(t\right)\right)-G_{h}\left(t\right)}{\tau_{h}},\\ E_{h}=\frac{RT}{F}\ln\frac{{\left[{Na}^{+}\right]}_{o}+5{\left[K^{+}\right]}_{o}}{{\left[{Na}^{+}\right]}_{V}+5{\left[K^{+}\right]}_{V}}, \end{array}$$

where *V*_S_ and *V*_V_ represent the membrane potential, *R*_S_ and *R*_V_ are the input resistance, *C*_S_ and *C*_V_ are the membrane capacitance, and *G*_h−S_ and *G*_h−V_ are the conductance of the HCN channels in the compartments of the soma and microvilli, respectively. ss*G*_h_ and τ_h_ are the steady-state conductance and opening/closing time constant (250 ms) of the HCN channels. *R*_i_ is the resistance between the two compartments. *E*_h_ is the reversal potential for *I*_h_, *E*_K_ = −97 mV, and *G*_hMax_, *V*_half_ and *S*_f_ are the maximal conductance, half-activation potential (−100 mV) and slope factor (11 mV) for *I*_h_, respectively. The mathematical model described by these formula can be represented by the equivalent circuit (**Figure 8B**). The Na^+^ concentration in the microvilli ([Na^+^]_V_) is expressed as follows:

$$\begin{array}{l} \frac{d{\left[{\text{Na}}^{+}\right]}_{\text{V}}}{dt}=-\frac{N_{0}P\left(t\right)i_{\text{Na}}\left(t\right)}{LF}-\frac{\left({\left[{\text{Na}}^{+}\right]}_{\text{V}}-{\left[Na^{+}\right]}_{\text{S}}\right)}{\tau }, \end{array}$$

where [Na^+^]_S_, *L* and τ are the Na^+^ concentration in the soma, the volume of the microvilli compartments, and the equalization time constant for the Na^+^ concentration between the soma and microvilli compartments, respectively. The value of [Na^+^]_V_ under the resting condition at −70 mV is equal to [Na^+^]_S_ (= [Na^+^]_i_). In addition to the Na^+^ microdomain model, we also simulated *I*_GluR_ with the *I*_h_ deactivation model, in which [Na^+^]_V_ remained constant (same as [Na^+^]_S_) and the *I*_h_ deactivation was caused by a large space-clamp error that was created by introducing a large resistance between the soma and microvillus compartments.

## Results

### Effects of 8-Br-cAMP on spike firings induced by AMPA puff application or current pulse injection

**Figure 1:**
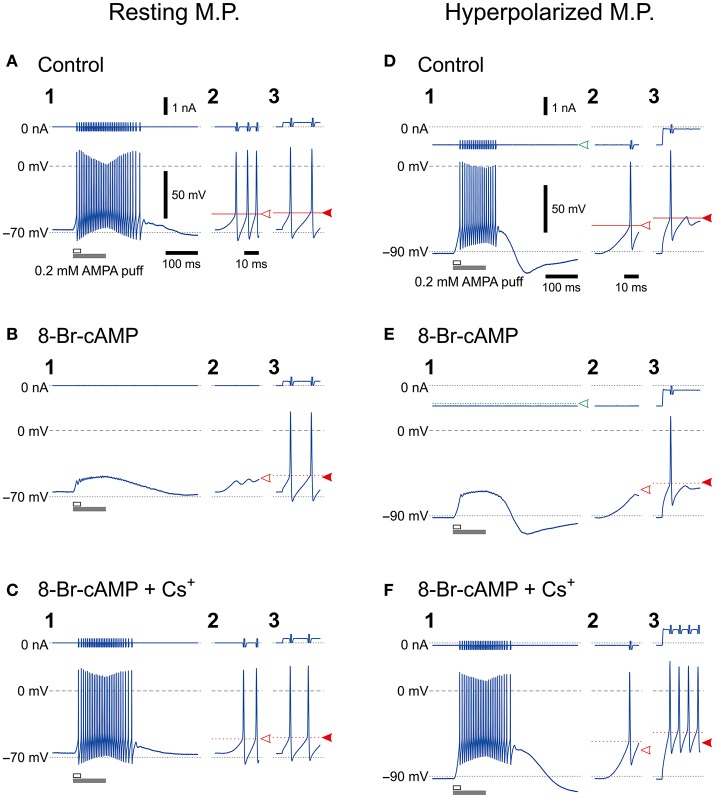
Effects of 8-Br-cAMP and Cs^+^ on spike firing induced by AMPA puff application or current pulse injection. *Bottom*, Membrane potential responses to a 100-ms puff of AMPA (**1**,**2**) or a 300-ms depolarizing current pulse (**3**) obtained before (**A**,**D**), during application of 8-Br-cAMP (**B**,**E**), and during the coapplication of 8-Br-cAMP and CsCl (**C**,**F**) under current-clamp conditions at baseline potentials of −70 mV (**A–C**) and −90 mV (**D–F**). Enlarged traces (**2**) seen during the respective time periods indicated with the open horizontal bars in **1**. *Top*, Membrane currents. Panels labeled with **3** show the responses to the current pulses only for approximately 14 ms from the pulse onsets. Spikes that were evoked by current pulses were not affected by the possible shunting effects of *I*_h_ brought about by 8-Br-cAMP, which was consistent with the effects of 8-Br-cGMP that enhances leak K^+^ currents as well as *I*_h_ (Supplementary Figure 1). The calibrations in **A1** also apply in all panels labeled with **1**. The time calibration in **A2** also applies in all panels labeled with **2** or **3**.

We previously demonstrated that HCN1/2 channels are expressed not only in cell membrane but also in microvilli together with Na^+^/K^+^ pump (Kang et al., 2004). To investigate the possible functional interactions between HCN and GluR channels during spike firing in MTN neurons, we first examined the effects of 8-Br-cAMP (an activator of cyclic nucleotide-gated channel) on the firing activities caused by a puff application (100 ms duration) of AMPA and current-pulse injections at a resting and a hyperpolarized membrane potentials (−70 and −90 mV, respectively) under the current-clamp condition. The mean resting membrane potential was −68.2 ± 3.4 mV (*n* = 11). The AMPA puff application induced high-frequency burst firings (Figures 1A1,D1) but caused no spike firings in the presence of 8-Br-cAMP, although 8-Br-cAMP slightly but significantly (^*^P < 0.001) depolarized the resting membrane potential (−65.4 ± 3.8 mV; *n* = 11) (Figures 1B1,E1). However, the burst firings were restored following the bath application of Cs^+^ (used as an *I*_h_ blocker) in addition to 8-Br-cAMP (Figure 1C1,F1), although Cs^+^ may also block K^+^ channels. In contrast, the bath application of 8-Br-cAMP did not affect the spike generation caused by injection of depolarizing current pulses despite the similar threshold for evoking the burst and the spike generation at the resting membrane potential (Figures 1A2–3,B2–3). On the other hand, when examined at −90 mV, which was brought about by increasing the negative DC level from −0.77 ± 0.30 to −0.97 ± 0.36 nA (*n* = 8), the threshold for inducing the burst by activation of GluR was lower than that for spikes evoked by the current pulse (Figures 1D2–3,E2–3). If the inhibition of spiking was due to the shunting effects of HCN channels, the spiking with the higher threshold would be more easily inhibited by the shunting effect, contrary to what was observed here. More importantly, 8-Br-cAMP never changed the current or voltage threshold for evoking spikes by injection of current pulses regardless of the baseline potentials of either −70 or −90 mV at which the current pulses were applied. This was also true for 8-bromoguanosine 3′,5′-cyclic monophosphate sodium salt (8-Br-cGMP; an activator of cyclic nucleotide-gated channel) that activates TASK1 leak K^+^ current as well as *I*_h_ (see Supplementary Figure 1). Given the activation of *I*_h_ by 8-Br-cAMP, these observations strongly suggest that, at least in MTN neurons, the shunting effects of *I*_h_ were not involved in the inhibition of the bursting by the activation of GluR, and the bursts appeared to be suppressed by a functional interaction between GluR and HCN channels. At the resting membrane potential (−70 mV), Cs^+^ application in the presence of 8-Br-cAMP restored the burst firing without changing the responses to current pulses (Figure 1C), whereas at −90 mV, Cs^+^ application caused stronger responses due to the blockade of *I*_h_ and *I*_K_, which had been more strongly activated at −90 mV than at −70 mV (Figure 1F). Cs^+^ application in addition to 8-Br-cAMP at −90 mV would have caused a further membrane hyperpolarization from −90 mV unless the negative DC level was decreased from −1.03 ± 0.38 nA to −0.56 ± 0.43 nA (*n* = 6).

### Effect of Cs^+^ on currents induced by glutamate puffs

**Figure 2:**
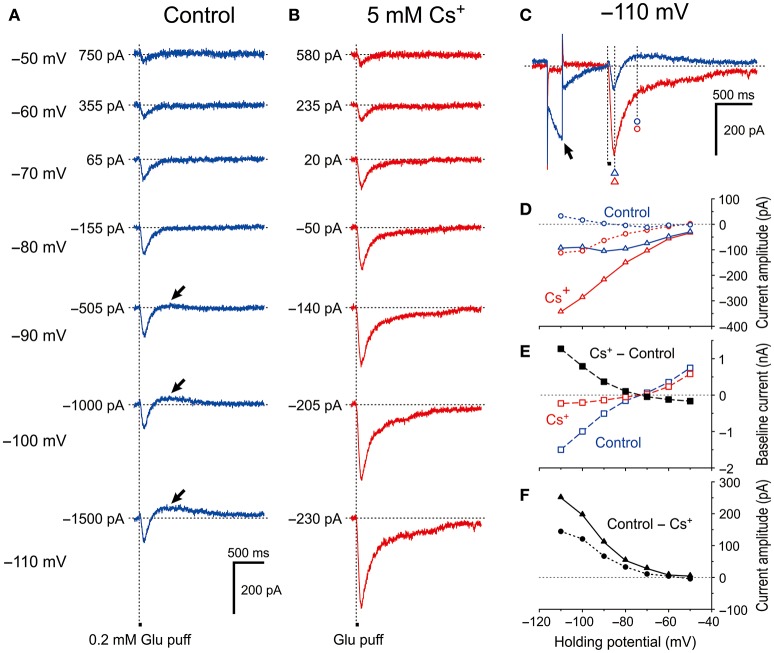
Voltage dependency of *I*_GluR_ and effects of Cs^+^ on *I*_GluR_. **(A)** Current responses to 50-ms puff applications of 0.2 mM glutamate (*I*_GluR_) recorded under the voltage-clamp condition at holding potentials ranging between −110 anfd −50 mV in MTN neurons. **(B)** Bath application of 5 mM Cs^+^ outwardly shifted the baseline current level, enhanced the inward component of *I*_GluR_ (IN-*I*_GluR_), and abolished the outward component of *I*_GluR_ (OT-*I*_GluR_) seen at membrane potentials below −80 mV in the absence of Cs^+^. **(C)** Current responses at −110 mV obtained in the absence (blue trace) and presence (red trace) of Cs^+^. The baseline current levels were aligned. **(D)** *I-V* relationships of *I*_GluR_ obtained in the absence (blue symbols) and the presence (red symbols) of Cs^+^. The triangles and circles represent the peak amplitudes of *I*_GluR_ and the amplitudes of *I*_GluR_ measured at 300 ms after the onset of the puff application of glutamate at which time *I*_GluR_ exhibited a peak value of the outward component at −110 mV, respectively. **(E)** *I-V* relationships of the baseline current obtained in the absence (blue squares) and the presence (red squares) of Cs^+^. The black squares represent the amplitudes of outward shifts of the baseline current at respective holding potentials obtained by subtraction of the control response (blue squares) from that recorded during Cs^+^ application (red squares). **(F)** *I-V* relationships of the Cs^+^-sensitive components of *I*_GluR_ that were obtained by subtraction of the response recorded during Cs^+^ application (red symbols) from the control (blue symbols) shown in **D**. The filled triangles and circles represent the amplitudes of Cs^+^-sensitive *I*_GluR_ measured at its peak and at 300 ms after the onset of puff, respectively.

To exclude the possible shunting effects of HCN currents (*I*_h_) or the effects of *I*_h_ deactivation, we further explored whether the HCN channels in MTN neurons can modify GluR-mediated currents under voltage-clamp conditions. We first tested the effect of blocking *I*_h_ with 5 mM extracellular Cs^+^, which is an effective blocker of *I*_h_ (Macri and Accili, 2004; Wu et al., 2012; Yang et al., 2015). We found that Cs^+^ enhanced the GluR currents (*I*_GluR_) in a highly voltage-dependent manner. In response to a short glutamate puff (0.2 mM, 50 ms duration), *I*_GluR_ was evoked at various holding potentials in the absence and presence of 5 mM Cs^+^ under the voltage-clamp condition (Figure 2). In the absence of Cs^+^, the amplitude of the *I*_GluR_ appeared to increase with membrane hyperpolarization up to −90 mV, whereas it was not increased but rather slightly decreased by further membrane hyperpolarization and was followed by an outward current that increased with membrane hyperpolarization (arrows, Figure 2A). In contrast, in the presence of Cs^+^, the *I*_GluR_ monotonically increased in amplitude with membrane hyperpolarization and was not followed by any outward currents (Figure 2B). These features are well illustrated in the *I*_GluR_*-V* relationship obtained before and during the bath application of Cs^+^. As the holding potential was hyperpolarized from −50 to −90 mV, the *I*_GluR_ measured at 95 ms after the puff application (Figure 2C) gradually increased (Figure 2A; blue open triangles, Figure 2D). However, at membrane potentials below −90 mV, the inward component of the *I*_GluR_ (IN-*I*_GluR_) was decreased and the outward component measured at 395 ms after the puff application (Figure 2C) emerged (OT-*I*_GluR_; arrows, Figure 2A; blue open triangles and circles, respectively, Figure 2D). Thus, with a negative shift of the holding potential, IN-*I*_GluR_ did not increase linearly despite the linear increase in the driving potential while OT-*I*_GluR_ became more prominent. In view of the emergence of OT-*I*_GluR_ and its increase with negative shifts of the holding potential, the IN-*I*_GluR_ may have been curtailed by some outward current that increases as the holding potential is negatively shifted.

After bath application of Cs^+^ (Figure 2B), both the *I-V* relationships of *I*_GluR_ measured at 95 and 395 ms after the puff application (Figure 2C) were almost linear (red open triangles and circles, respectively, Figure 2D). This linear *I-V* relationship of *I*_GluR_ was invariably observed following Cs^+^ application in the 11 examined MTN neurons. Consequently, the amplitudes of the IN-*I*_GluR_ at −70 mV were significantly increased by 26% ± 19% (^*^P < 0.002). Concomitantly, Cs^+^ abolished the *I*_h_ that was produced by a hyperpolarizing prepulse (arrow, Figure 2C), which is consistent with the outward shift of the baseline current that reflects the instantaneous or standing *I*_h_ at the respective membrane potentials (Figure 2E). The Cs^+^-sensitive outward component of *I*_GluR_ (black filled triangles and circles, Figure 2F) that was obtained by subtraction of the response recorded after Cs^+^ application from the control revealed a voltage dependence similar to that of *I*_h_. Giving the sensitivity of *I*_h_ to Cs^+^, this *I-V* relationship (black filled triangles, Figure 2F) suggests that *I*_h_ was involved in the apparent inhibition of IN-*I*_GluR_ and in the generation of OT-*I*_GluR_.

### Effect of *I*_h_ activation with 8-Br-cAMP on *I*_GluR_

**Figure 3:**
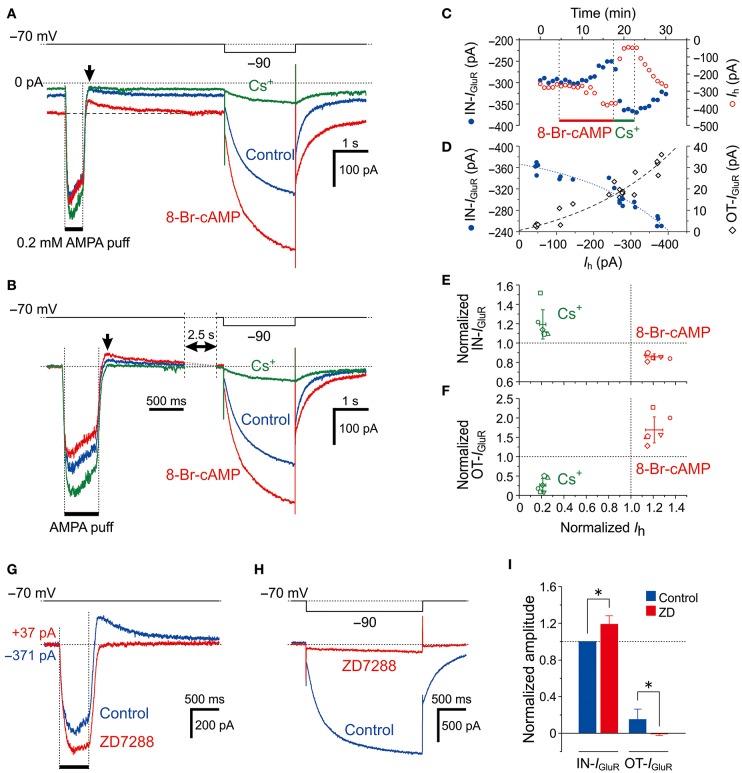
Effects of *I*_h_ activation by 8-Br-cAMP on *I*_GluR_. **(A**,**B)** Current responses to a 500-ms puff application of 0.2 mM AMPA and a hyperpolarizing pulse (−20 mV, 500 ms) recorded before (blue traces), during the application of 8-Br-cAMP (red traces), and during the application of Cs^+^ (green traces) under the voltage-clamp condition at −70 mV (**A**). The baseline current levels were aligned (**B**). The arrows indicate the outward components of *I*_GluR_ (OT-*I*_GluR_). **(C)** Plot of the amplitudes of the inward component of *I*_GluR_ (IN-*I*_GluR_; blue filled circles) and the *I*_h_ (red open circles) against time during applications of 8-Br-cAMP and Cs^+^. Note the reciprocal changes in the amplitudes of IN-*I*_GluR_ and *I*_h_. **(D)** Plot of the amplitudes of IN-*I*_GluR_ (blue filled circles) and OT-*I*_GluR_ (black open diamonds) against those of *I*_h_ obtained before and during 8-Br-cAMP and Cs^+^ applications. Note the negative correlation between the amplitudes of *I*_h_ and the IN-*I*_GluR_ and the positive correlation between the amplitudes of *I*_h_ and the OT-*I*_GluR_. **(E**,**F)** Relationship between the normalized amplitudes (mean ± SD) of the IN-*I*_GluR_ (**E**) and OT-*I*_GluR_ (**F**) and that of *I*_h_ observed during 8-Br-cAMP (red symbols) and Cs^+^ (green symbols) applications in six MTN neurons (*n* = 6). A set of four (red and green, **E**,**F**) symbols with the same shape represent data obtained from a single neuron. 8-Br-cAMP: *I*_h_, 1.21 ± 0.08 (*P < 0.002); IN-*I*_GluR_, 0.86 ± 0.03 (*P < 0.001); OT-*I*_GluR_, 1.69 ± 0.33 (*P < 0.006). Cs^+^: *I*_h_, 0.21 ± 0.03 (*P < 0.001); IN-*I*_GluR_, 1.19 ± 0.15 (*P < 0.04); OT-*I*_GluR_, 0.26 ± 0.17 (*P < 0.001). **(G**,**H)** Current responses to a 500-ms puff application of 0.2 mM AMPA (**G**) and a negative voltage pulse (−20 mV, 2 s) (**H**) recorded before (blue traces) and during the application of ZD7288 (red traces). The baseline current levels were aligned. **(I)** Mean (± SD) normalized amplitudes of the IN-*I*_GluR_ and OT-*I*_GluR_ before (blue columns) and during the application of ZD7288 (red columns) (*n* = 6). *: *P* < 0.05.

MTN neurons receive serotonergic synaptic inputs (Tanaka and Chandler, 2006) that activate 5-HT receptors to stimulate the production of cAMP, which in turn activates HCN channels through the binding with a cyclic nucleotide-binding domain (Wainger et al., 2001; Wang et al., 2007). To further investigate the involvement of HCN channels in the inhibition of *I*_GluR_, we next examined the effects of 0.5 mM 8-Br-cAMP on the *I*_GluR_ evoked at −70 mV in response to a 500-ms puff applications of 0.2 mM AMPA (Figure 3). Bath application of 8-Br-cAMP shifted the baseline current inwardly from −149 ± 103 pA to −250 ± 148 pA (*n* = 6, ^*^P < 0.007) and decreased the amplitude of the IN-*I*_GluR_ while increasing the amplitude of the following OT-*I*_GluR_ (blue and red traces, Figure 3A) concomitantly with an increase in *I*_h_ that was evoked by a negative pulse, as revealed by the superimposed traces aligned with their baseline levels (Figure 3B). In contrast, bath application of Cs^+^ right after the 8-Br-cAMP session shifted the baseline current outwardly to −76 ± 84 pA (*n* = 6, ^*^P < 0.02) and increased the amplitude of the IN-*I*_GluR_ but completely abolished the OT-*I*_GluR_ concomitant with a marked inhibition of *I*_h_ (green traces, Figures 3A,B). These reciprocal changes between the IN-*I*_GluR_ and *I*_h_ amplitudes that were observed during 8-Br-cAMP and Cs^+^ applications were represented by plotting the amplitudes of the IN-*I*_GluR_ and *I*_h_ against time (blue and red circles, respectively, Figure 3C). Subsequently, plotting the amplitudes of the IN-*I*_GluR_ (blue filled circles) and OT-*I*_GluR_ (black open diamonds) against the amplitudes of the *I*_h_ revealed significantly negative (#*P* < 0.001, *r* = −0.96) and positive correlations (#*P* < 0.001, *r* = 0.95), respectively (Figure 3D). The inverse relationship of the normalized amplitudes between the *I*_h_ and the IN-*I*_GluR_ and the proportional relationship between the *I*_h_ and the OT-*I*_GluR_ were obtained in six MTN neurons following bath applications of 8-Br-cAMP and Cs^+^ (Figures 3E and F, respectively). The 8-Br-cAMP significantly decreased the IN-*I*_GluR_ (^*^P < 0.001) but increased the OT-*I*_GluR_ (^*^P < 0.006) concomitant with increases in *I*_h_ (^*^P < 0.002) (red symbols, *n* = 6; Figures 3E,F). In contrast, Cs^+^ significantly increased IN-*I*_GluR_ (^*^P < 0.006) but decreased OT-*I*_GluR_ (^*^P < 0.001) concomitant with decreases in *I*_h_ (^*^P < 0.001) (green symbols, *n* = 6; Figures 3E,F). Although Cs^+^ may block various K^+^ currents as well as the *I*_h_, the inhibitory effect of Cs^+^ on K^+^ currents is very small at −70 mV. Indeed, consistent with these observations made with Cs^+^, the abolishment of the *I*_h_ by ZD7288 (Figure 3H) also significantly increased the amplitude of the IN-*I*_GluR_ and concomitantly abolished the OT-*I*_GluR_ completely (Figures 3G,I). These observations suggest that IN-*I*_GluR_ was curtailed by an apparent outward current that presumably flowed through the HCN channels and emerged as OT-*I*_GluR_ after the closure of the GluR channels at the offset of the AMPA puff.

Provided that Na^+^ ions flow intracellularly during *I*_GluR_ and accumulate in a microdomain of spine-like microvilli, the reversal potential for *I*_h_ (*E*_h_) should be transiently shifted in the negative direction resulting in a reduction of the driving potential of inward *I*_h_, which in turn would shift the baseline inward *I*_h_ at −70 mV in the outward direction. Then, the IN-*I*_GluR_ may be decreased due to the transient outward shift of the baseline *I*_h_, and the OT-*I*_GluR_ may become apparent following the cessation of the IN-*I*_GluR_ at the puff offset because the baseline *I*_h_ is likely to recover slowly following the extrusion of Na^+^ ions from the microdomain. The outward shift of the baseline *I*_h_ during *I*_GluR_ through the accumulation of Na^+^ ions may become larger as the conductance of HCN channels is increased by 8-Br-cAMP even if the reduction of the driving potential of *I*_h_ remains the same. Therefore, we hypothesized that 8-Br-cAMP decreases IN-*I*_GluR_ and increases OT-*I*_GluR_ by further outwardly shifting the baseline *I*_h_ at −70 mV during *I*_GluR_, which is consistent with the opposite effects of Cs^+^/ZD7288 on *I*_GluR_.

### Differential effects of ouabain on *I*_h_ and *I*_GluR_

**Figure 4:**
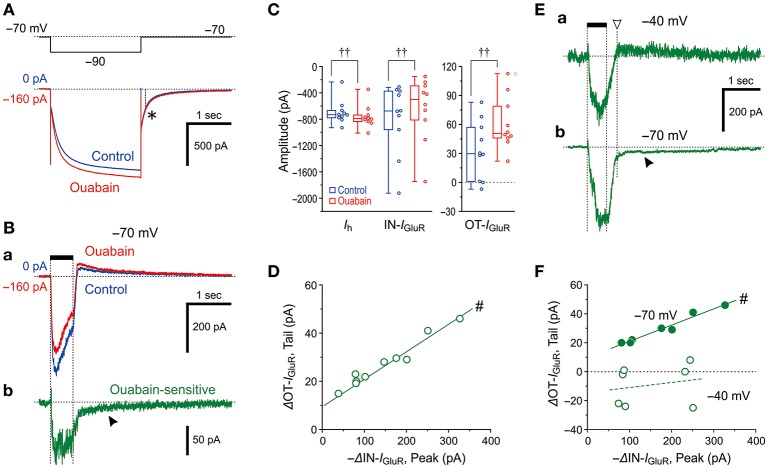
Effects of a Na^+^/K^+^ pump inhibitor on *I*_GluR_. **(A**,**B)** Current responses to a negative voltage pulse (−20 mV, 2 s) **(A)** and a 500-ms puff application of 0.2 mM AMPA **(Ba)** recorded before (blue traces) and during the application of 50 μM ouabain (red traces). The ouabain-sensitive *I*_GluR_, acquired by subtraction of the red trace from the blue trace shown in **Ba (Bb)**. Note the presence of a slow tail component, reflecting an enhancement of OT-*I*_GluR_ (arrowhead). The baseline current levels were aligned. The asterisk denotes the unchanged tail current before and during the application of ouabain. **(C)** Box-and-whisker plots represent the amplitudes of the steady-state *I*_h_, IN-*I*_GluR_, and OT-*I*_GluR_ obtained before (blue) and after 50 μM ouabain application (red). ††: *P* < 0.01 (Wilcoxon signed-rank test). **(D)** Plot of the increase in amplitude of OT-*I*_GluR_ against the decreases in the amplitudes of IN-*I*_GluR_ following ouabain application. #: *P* < 0.05 (Pearson correlation coefficient). **(E)** The ouabain-sensitive *I*_GluR_ at −40 mV (upper trace) and at −70 mV (lower trace). Note the absence and presence (filled arrowhead) of slow tail component that reflects an enhancement of OT-*I*_GluR_. The amplitude of OT-*I*_GluR_ was measured at the time indicated by downward open arrowhead. **(F)** Plot of changes in amplitude of OT-*I*_GluR_ against decreases in amplitudes of IN-*I*_GluR_ at −70 mV and −40 mV. Note the presence and absence of significant correlation between the two amplitudes, at −70 mV and −40 mV, respectively. #: *P* < 0.05 (Pearson correlation coefficient).

Because the Na^+^/K^+^ pump and HCN share a Na^+^ microdomain in the spine-like microvilli of MTN neurons as we previously reported (Kang et al., 2004), we next compared the effects of ouabain (Na^+^/K^+^ pump inhibitor) on *I*_h_ and *I*_GluR_ evoked at −70 mV. Ouabain shifted the baseline current in the inward direction and increased the amplitudes of the inward *I*_h_ evoked by a negative voltage pulse to −90 mV as a result of the suppression of the outward Na^+^/K^+^ pump-mediated current that might have been induced in response to the activation of *I*_h_ (Figure 4A). This observation is consistent with our previous study (Kang et al., 2004). In contrast, ouabain did not increase but rather decreased the IN-*I*_GluR_ whereas it increased the OT-*I*_GluR_ (Figure 4Ba; also see Figure 4C) concomitant with an apparent enhancement of *I*_h_ (Figure 4A). Thus, the ouabain sensitive current which was acquired by subtraction of the response obtained after application of ouabain from the control response was composed of the ΔIN-*I*_GluR_ and the slow inward tail-I as the ΔOT-*I*_GluR_ (Figure 4Bb). In the 10 MTN neurons examined, the inhibition of Na^+^/K^+^ pump-mediated current with 50 μM ouabain significantly shifted the baseline current in the inward direction [from −25 (IQR 29) pA to −171 (IQR 44) pA, †*P* < 0.006, *n* = 10] and increased *I*_h_ [from −732 (IQR 94) pA to −791 (IQR 83) pA, †*P* < 0.006, *n* = 10], whereas it significantly decreased the amplitudes of the IN-*I*_GluR_ at −70 mV from −676 (IQR 517) pA to −499 (IQR 456) pA (†*P* < 0.006, *n* = 10) and increased the amplitudes of the OT-*I*_GluR_ at −70 mV from 30 (IQR 46) pA to 51 (IQR 27) pA (†*P* < 0.006, *n* = 10; Figure 4C).

Such differential effects of ouabain on HCN and GluR would indicate that Na^+^ influx through HCN channels immediately and markedly activates Na^+^/K^+^ pumps, whereas Na^+^ influx through GluR would neither immediately nor markedly activate Na^+^/K^+^ pumps. Then, the apparent suppression of *I*_GluR_ is either brought about directly by an accumulation of Na^+^ that causes the reduction of the driving potential of *I*_GluR_ or caused by an enhancement of *I*_h_ as a result of the inhibition of Na^+^/K^+^ pump by ouabain, in a manner similar to the case with 8-Br-cAMP. Because the OT-*I*_GluR_ was also enhanced by ouabain, the inhibition of IN-*I*_GluR_ by ouabain was at least partly due to the generation of outward current mediated by a transient reduction of the enhanced (inwardly shifted) baseline *I*_h_ by ouabain during *I*_GluR_ that might have led to the generation of OT-*I*_GluR_. Indeed, there was a significant positive correlation between the decrease in IN-*I*_GluR_ and the increase in OT-*I*_GluR_ (#*P* < 0.001, *r* = 0.98, *n* = 10; Figure 4D). Next, we aimed to examine if there is *I*_h_-independent decrease in *I*_GluR_ following ouabain application at −40 mV at which *I*_h_ is not active at all. As revealed by the ouabain sensitive component of *I*_GluR_ at −40 mV, which was acquired by subtraction of *I*_GluR_ obtained after ouabain application from that of the control (Figure 4Ea), ouabain decreased *I*_GluR_ evoked at −40 mV but did not generate the slow inward tail component in contrast to the ouabain sensitive *I*_GluR_ at −70 mV (Figure 4Eb). This also suggests that the functional interaction between GluR and Na^+^/K^+^ pump is very weak if any and the effect of Na^+^ accumulation on *I*_GluR_ in microvilli overcame the interaction if any. As revealed by the presence or absence of the ouabain sensitive slow inward tail component of *I*_GluR_ (Figures 4Ea,b), *I*_GluR_ inhibition by ouabain at −70 mV was invariably accompanied by the enhancement of OT-I_GluR_, whereas *I*_GluR_ inhibition by ouabain at −40 mV was not accompanied by enhancement of OT-I_GluR_. Indeed, there was a significant positive correlation between the decrease in IN-*I*_GluR_ and the increase in OT-*I*_GluR_ when examined at −70 mV (#*P* < 0.001, *r* = 0.98, *n* = 7) whereas no significant correlation between the decrease in IN-*I*_GluR_ and the changes in OT-*I*_GluR_ when examined at −40 mV in the same MTN neurons (#*P* > 0.6, *r* = 0.21, *n* = 7; Figure 4F). Thus, even in the absence of HCN activity, GluR activation did not apparently stimulate Na^+^/K^+^ pump, suggesting that the Euclidean distance between Na^+^/K^+^ pump and GluR is much larger than that between Na^+^/K^+^ pump and HCN channels.

These observations and notions suggest that Na^+^ homeostasis around active HCN channels is strictly regulated by Na^+^/K^+^ pump as long as GluR is not activated, whereas the homeostasis around active GluR is not regulated by Na^+^/K^+^ pump regardless of the activity of HCN channels. Indeed, ouabain increased the *I*_h_ amplitude but did not significantly (†*P* > 0.1, *n* = 7) increase its tail current (asterisk), as the amplitude measured 0.1 sec after the offset of the negative command pulse to −90 mV (interrupted line) was slightly changed from −187 ± 67 pA to −195 ± 67 pA following ouabain application (Figure 4A). This suggests the negative shift of the reversal potential of *I*_h_ due to the accumulation of Na^+^ through the breakdown of Na^+^ homeostasis around HCN channels by ouabain (see Discussion). This finding is in contrast to the case with 8-Br-cAMP (compare Figures 3A,B, 4A). Although the negative shift of *E*_h_ has not been reported, the activity-dependent shift of the reversal potential is not unusual for ligand gated channels such as GABA_A_ (Fiumelli et al., 2005) or glycine (Kim and Trussell, 2009) receptor channels.

### Effects of the change in the reversal potential for *I*_h_ on *I*_GluR_

**Figure 5:**
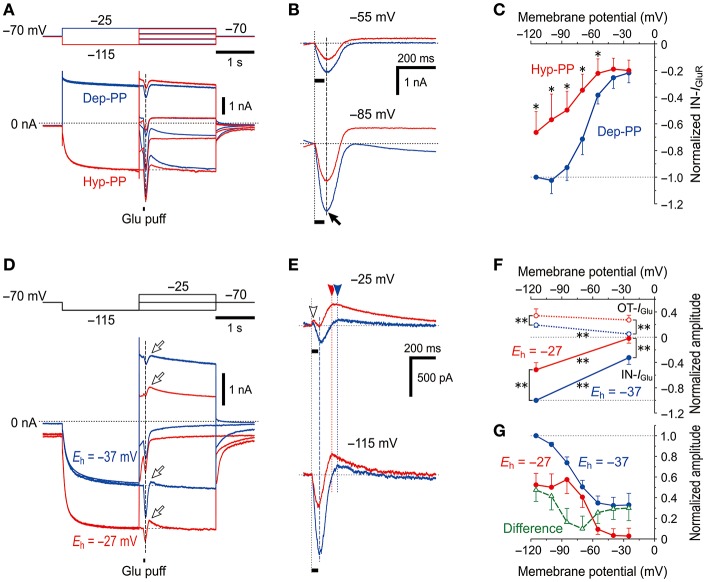
Voltage-dependent inhibitory effects of *I*_h_ on *I*_GluR_ **(A)** *I*_GluR_ responses evoked at various membrane potentials following either the activation of *I*_h_ by a hyperpolarizing prepulse (Hyp-PP; red traces) or the deactivation of *I*_h_ by a depolarizing prepulse (Dep-PP; blue traces). **(B)** Superimposed *I*_GluR_ responses evoked at −55 (top) and −85 mV (bottom) showing the decrease in the amplitude of *I*_GluR_ following the activation of *I*_h_. **(C)** *I-V* relationships of *I*_GluR_ obtained following hyperpolarizing (red) and depolarizing prepulses (blue circles). The amplitudes of *I*_GluR_ were normalized to the amplitude of *I*_GluR_ at −120 mV following a depolarizing prepulse. *P < 0.05. **(D)** Sample traces of *I*_GluR_ responses evoked at various membrane potentials following activation of *I*_h_ by a hyperpolarizing prepulse obtained in the different extracellular solutions in which *E*_h_ is either −37 (blue) or −27 mV (red traces). **(E)** Superimposed *I*_GluR_ responses evoked at −25 (top) and −115 mV (bottom) showing the decrease in the amplitude of *I*_GluR_ following the activation of *I*_h_ with *E*_h_ of −27 mV compared with *E*_h_ of −37 mV. **(F)** *I-V* relationships of OT- and IN-*I*_GluR_ obtained in the respective conditions. **P < 0.01. **(G)** The amplitudes of the IN-*I*_GluR_ normalized to that at −115 mV under the condition of *E*_h_ = −37 mV. Note the U-shaped voltage-dependent inhibition of IN-*I*_GluR_ following the increase in the driving potential for *I*_h_ (green open triangles).

*I*_GluR_ was evoked at the respective membrane potentials that ranged between −115 and −25 mV after the depolarizing (−25 mV; blue traces) or hyperpolarizing (−115 mV; red traces) prepulse that largely deactivated or activated *I*_h_, respectively (Figure 5A). The amplitudes of the IN-*I*_GluR_ obtained at the respective membrane potentials after the hyperpolarizing prepulse (red traces) were smaller than those obtained after the depolarizing prepulse (blue traces, Figure 5B). As revealed in the plot of the amplitudes of the IN-*I*_GluR_ against the membrane potentials (Figure 5C), the IN-*I*_GluR_ obtained below −55 mV after the hyperpolarizing prepulse was significantly (*n* = 5, ^*^P < 0.05) smaller than those obtained after the depolarizing prepulse. In this experiment, an inhibition of IN-*I*_GluR_ was observed following increases in the conductance of the HCN channels.

In the next experiment, *I*_h_ was increased by increasing the driving potential without changing the conductance of HCN channels to directly clarify whether the inhibitory effects of *I*_h_ on glutamate responses were due to its shunting effect or the functional interaction between the two channels. [K^+^]_o_ was increased from 3 to 21 mM by replacing 18 mM Na^+^ with equimolar K^+^ to shift the reversal potential for K^+^ currents (*E*_K_) from −97 to −47 mV and to shift the *E*_h_ from −37 to −27 mV while leaving the reversal potential for *I*_GluR_ unchanged due to the equal permeability of GluR channels to K^+^ and Na^+^. Following an increase in [K^+^]_o_, the baseline current at −70 mV shifted inwardly, which suggests that the standing *I*_h_ reflected in the baseline current was increased by increasing the driving potential by 10 mV together with a generation of an inward leak K^+^ current. Concomitantly, the IN-*I*_GluR_ was clearly decreased while the OT-*I*_GluR_ (arrows) was clearly enhanced both at −25 and −115 mV (Figure 5D; also see Figure 5E). The amplitudes of IN-*I*_GluR_ (filled circles) and OT-*I*_GluR_ (open circles) obtained under the condition of *E*_h_ = −27 mV (red symbols) were significantly smaller (−25 mV, ^*^P < 0.001 and −115 mV, ^*^P < 0.001) and larger (−25 mV, ^*^P < 0.001 and −115 mV, ^*^P < 0.001), respectively, than those obtained under the control condition of *E*_h_ = −37 mV (*n* = 7; blue symbols, Figure 5F). The normalized decrease in the amplitude of IN-*I*_GluR_ following the shift of *E*_h_ from −37 mV (blue filled circles) to −27 mV (red filled circles) was found to have a U-shaped voltage dependence with the minimal value at −70 to −60 mV (green open triangles, Figure 5G), although such an estimation for OT-*I*_GluR_ was difficult due to its slower time-to-peak and the differential relaxation of *I*_h_ between the responses under the two different conditions of *E*_h_.

Thus, without a conductance increase in HCN channels but with a positive shift of *E*_h_ by a [K^+^]_o_ increase, *I*_GluR_ was more strongly canceled. Simple voltage-dependent deactivation of *I*_h_ is not compatible with the U-shaped voltage dependence of inhibition of IN-*I*_GluR_ because the deactivation of the possible outward baseline *I*_h_ generated at −25 mV would result in an increase in the IN-*I*_GluR_ and a decrease in the OT-*I*_GluR_. These observations and notions clearly indicate that *I*_GluR_ was suppressed neither by the shunting effects of *I*_h_ nor by the deactivation of *I*_h_, but was rather canceled by a decrease in the inward baseline *I*_h_ or an increase in the outward baseline *I*_h_ that was induced during *I*_GluR_ depending on the membrane potential at which *I*_GluR_ was evoked (Figure 5G).

A possible negative shift of *E*_h_ due to accumulation of Na^+^ in the microvillus during *I*_GluR_ can cause a decrease in the inward baseline *I*_h_ at −115 mV and an increase in the outward baseline *I*_h_ at −25 mV, both of which should result in the decrease in IN-*I*_GluR_ and increase in OT-*I*_GluR_. This assumption is strongly supported by the U-shaped voltage dependence of the decrease in the amplitude of IN-*I*_GluR_ (Figure 5G). Taken together, it is likely that *I*_GluR_ can be decreased either by decreasing the driving potential for inward *I*_h_ or by increasing the driving potential for outward *I*_h_ depending on the holding potential, through the accumulation of Na^+^ ions in the microvillus which serves as a Na^+^ microdomain.

### Na^+^ accumulation in the microvilli in response to the activation of GluR

Because a transient negative shift of the reversal potential for *I*_h_ is likely to be caused by a transient increase in Na^+^ concentration in microdomains following the activation of GluR, we next addressed whether Na^+^ concentration transiently increases in the microvilli in response to activation of GluR using a Na^+^ indicator, Sodium Green.

**Figure 6:**
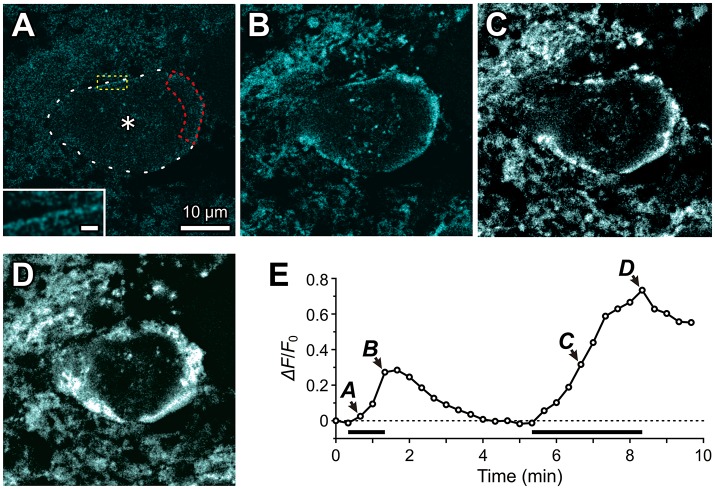
Fluorescence imaging of Na^+^ accumulation in the microvilli of MTN neurons using Sodium Green tetraacetate. Fluorescence images of Na^+^ concentration were captured every 20 sec in response to 1 mM glutamate application for 1 min and for 3 min in a Sodium Green-loaded MTN neuron. The respective fluorescence images **(A–D)** were obtained by subtraction of the frame 2 response from those of the respective frames (3rd, 5th, 21st, and 26th). **(A)** Twenty seconds after the onset of a 1 min glutamate application, apparent Na^+^ concentration increases can hardly be observed, but with a close look (*inset*) very thin or small dot-like fluorescence images can be observed along the contour of the MTN neuron (interrupted line). The region encircled with the red interrupted line represents the ROI. Scale bar in *inset*: 1 μm. **(B)** A Na^+^ concentration increase seen just beneath the plasma membrane or microvilli of a Sodium Green-loaded MTN neurons at the offset of a 1-min glutamate application. **(C**,**D)** A larger Na^+^ concentration increase captured at 80 sec **(C)** and 180 sec **(D)** after the onset of a 3-min glutamate application. **(E)** A time course of Na^+^ accumulation evoked twice in response to two successive application of 1 mM glutamate for 1 and 3 min separated by 4 min. The arrowheads with the letters **A–D** indicate the averaged fluorescence intensity in the ROI in the images **A–D**, respectively. The horizontal bars indicate the timing and duration of the glutamate application.

Using a confocal microscope, we performed fluorescence measurements of Na^+^ concentration changes in Sodium Green-loaded MTN neurons in response to the bath application of 1 mM glutamate (Figure 6). The Na^+^ concentration was gradually increased only just beneath the plasma membrane or presumably in the microvilli (Figure 6B), while the cytoplasm did not exhibit any marked increases in Na^+^ concentration in an MTN neuron (asterisk, Figure 6A). The first glutamate application for 1 min caused a Na^+^ transient that exhibited a more than 50% decay within 80 s from the offset of the glutamate application (Figures 6A,B,E). In contrast, the second application of glutamate for 3 min caused a larger increase in Na^+^ concentration not only in microvilli but also partly in the cytoplasm (Figures 6C,D), which exhibited a less than 25% decay after 80 sec from the offset of the glutamate application (Figure 6E), suggesting that the Na^+^/K^+^ pump activity was involved in the regulation of the decay time course in a manner dependent on its availability that was inversely proportional to the Na^+^ concentration. This notion further suggests that successive glutamatergic synaptic inputs may be more strongly depressed by HCN activity, as has been reported previously (Magee, 1999; Carr et al., 2007). In a total of seven MTN neurons, 1 min of glutamate application increased the Δ*F*/*F*_0_ by 31 ± 12% just beneath the cell membrane or microvilli. It should be noted that the rate constant for Na^+^ binding may be too slow to detect the rapid and large increase in Na^+^ in microvilli (Figure 6E; see section Materials and Methods).

### Glutamatergic synapses on the microvilli expressing HCN2 channels in MTN neurons

**Figure 7:**
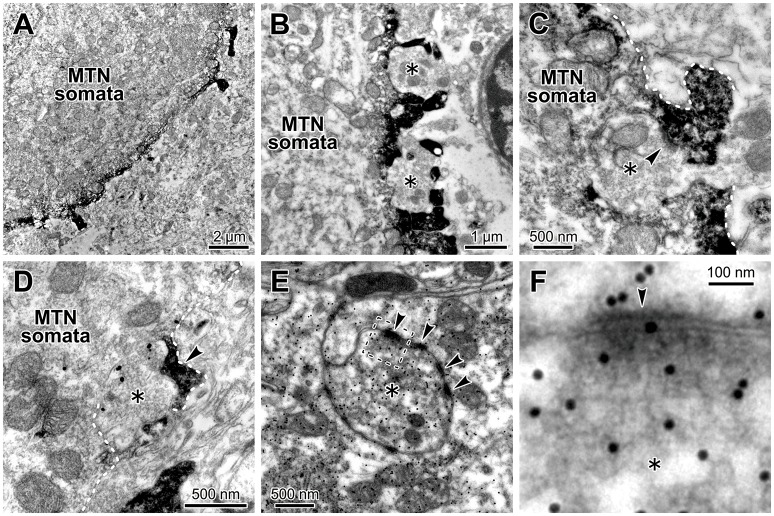
Glutamatergic axon terminals contact with HCN2-immunopositive small processes of the MTN neurons. **(A**,**B)** HCN2 immunoreactivity was localized in the small process and periphery of the MTN neuron. A terminal bouton (asterisk) contacted with an HCN2-immunopositive small process (arrowhead) of the MTN neuron **(C)**. **(C)** The white dotted line demarcates the border of the small process. HCN2 immunoreactivity was observable as an electron-dense immunoreactive product. **(D)** A VGLUT2-immunopositive axon terminal (gold particles, asterisk) contacted with an HCN2-immunopositive small process (arrowhead) of the soma of the MTN neuron. The white dotted line demarcates the border of the small process. **(E**,**F)** Terminal boutons (asterisks) containing round vesicles that were immunopositive for an anti-glutamate antibody **(E)** contacted with the processes of the MTN neurons. Note that the glutamatergic terminal (silver grains) formed asymmetrical synaptic contacts (arrowheads) with a process. The area enclosed by a rectangle in **E** is enlarged in **F**.

Because we already demonstrated that in juvenile rats HCN1/2 are expressed in microvilli (Kang et al., 2004), we next confirmed that glutamatergic synapses are colocalized with HCN channels in microvilli of MTN neurons in adult rats. Electron-microscopic immunohistochemistry revealed that HCN2 immunoreactivity was observable as an electron-dense product that was localized in the spines of the MTN neuron (Figures 7A,B) and that a terminal bouton (asterisk) made a synaptic contact (arrowhead) with an HCN2-immunopositive spine of the MTN neuron (Figure 7C). Double immunostaining for HCN2 and vesicular glutamate transporter 2 (VGLUT2) revealed that a VGLUT2-immunopositive axon terminal (asterisk) made a synaptic contact on an HCN2-immunopositive spine (arrowhead) of the soma of the MTN neuron (Figure 7D). Furthermore, a terminal bouton (asterisk) of a glutamate-immunopositive axon formed asymmetrical synaptic contacts (arrowhead) with an HCN2-immunopositive spine (Figures 7E,F). These observations indicated that the glutamatergic axon arising from central neurons but not primary afferents (Pang et al., 2009) made synaptic contacts on the HCN-immunopositive spine that directly protrudes from the round shaped soma of the MTN neuron. Because it is known that in MTN neurons, HCN channels (Tanaka et al., 2003) and various synaptic inputs including glutamatergic one (Paik et al., 2012) are developmentally mature by PND 13 at the latest, these data obtained from adult rats can be extrapolated to juvenile rats at PND 13–18. Taking our previous study (Kang et al., 2004) into consideration together with the present morphological findings, the present electrophysiological findings obtained in juvenile rats can be extended to adult rats, eliminating the possibility that the functional interaction between HCN and GluR channels is a transient phenomenon accompanying the postnatal development of MTN neurons.

### A mathematical model of the *I*_GluR_ inhibition by the activity of HCN channels

**Figure 8:**
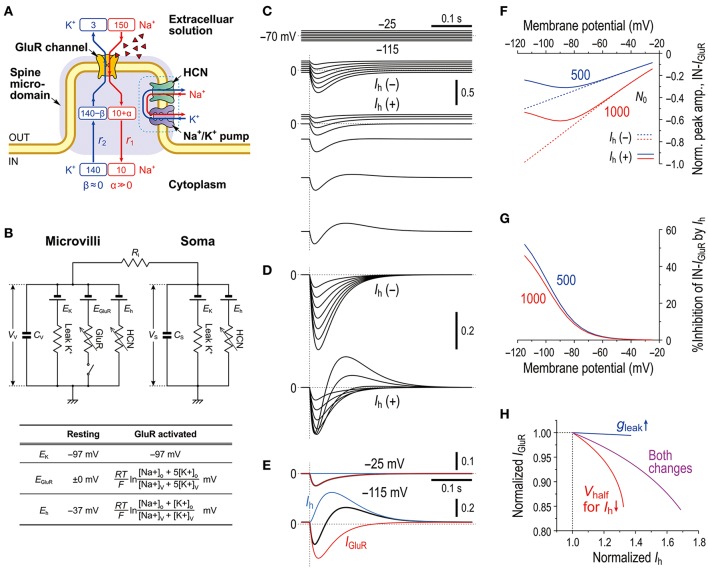
Mathematical modeling of *I*_GluR_ inhibition with a negative shift of *E*_h_ through Na^+^ accumulation in the microvilli mimics the experimental results. **(A)** A schematic diagram showing a microvillus in which GluR and HCN were colocalized together with a Na^+^/K^+^ pump that has a functional coupling with the HCN channel. Only the Na^+^ concentration can be transiently increased via the activity of GluR, which consequently affects the reversal potentials for GluR and HCN channels. The HCN activity causes no changes in Na^+^ in a microvillus due to the coupling with Na^+^/K^+^ pump. The K^+^ concentration in the microvillus remains constant because K^+^ efflux through GluR is instantaneously and easily compensated from the soma. **(B)** Equivalent circuit of the mathematical model. **(C)** Simulated current responses to a glutamate puff obtained at the respective holding potentials (top panels, **C**) in the absence of HCN [*I*_h_(–)] or in the presence of HCN [*I*_h_(+)]. Maximum numbers of activated GluR channels *N*_0_ = 500. [Na^+^]_o_ = 150 × 10^−3^ (M), [K^+^]_o_ = 3 × 10^−3^ (M), [Na^+^]_i_ = 14 × 10^−3^ (M), [K^+^]_i_ = 141 × 10^−3^ (M), *T* = 293.15 (K), τ_1_ = 20 × 10^−3^ (s), τ_2_ = 40 × 10^−3^ (s), *R*_S_ = 50 × 10^6^ (Ω), *R*_V_ = 400 × 10^6^ (Ω), *R*_i_ = 5 × 10^5^ (Ω), *C*_S_ = 40 × 10^−12^ (F), *C*_V_ = 5 × 10^−12^ (F), *G*_hMax_ for *G*_h−S_ = 3.5 × 10^−9^ (S), *G*_hMax_ for *G*_h−V_ = 35 × 10^−9^ (S), *L* = 210 × 10^−18^ (m^3^), and τ = 50 × 10^−3^ (s). **(D)** Superimposed traces of the simulated *I*_GluR_ shown in **C**. **(E)** The current responses obtained at −25 and −115 mV shown in [**D**, *I*_h_(+)] were separated into *I*_h_ and *I*_GluR_ components (blue and red traces, respectively). **(F)** Non-linear (solid curves; under *I*_h_) and linear (interrupted curves under no *I*_h_) *I-V* relationships of the peak amplitudes of IN-*I*_GluR_ normalized to that obtained at −115 mV under conditions of no *I*_h_ with *N*_0_ = 1,000 (red interrupted lines). The blue and red curves were obtained under the conditions of *N*_0_ = 500 and 1,000, respectively. **(G)** Voltage-dependent relative inhibitions of the peak IN-*I*_GluR_ with the respective *N*_0_ calculated from **(F)**. **(H)** The relationship between the normalized amplitudes of *I*_h_ and *I*_GluR_ seen following the simultaneous increase in *g*_Leak_ and the negative shift of *V*_half_ (magenta curve) and following the sole negative shift of *V*_half_ (red curve). The magenta and red curves simulated the effects of 8-Br-cAMP and 8-Br-cGMP on *I*_GluR_. In the case of the sole increase in *g*_Leak_ (blue curve), leak K^+^ current was reflected in an instantaneous component of *I*_h_. These results were experimentally confirmed (Supplementary Figure 2).

As we previously reported the bidirectional interaction between HCN and Na^+^/K^+^ pump co-localized in the same microvillus in MTN neurons (Kang et al., 2004), Na^+^ influx/K^+^ efflux through HCN channels into the microvillus would not affect their own reversal potentials (*E*_h_) due to the strict regulation of Na^+^/K^+^ homeostasis around the active HCN channels by the Na^+^/K^+^ pump (see Discussion section). This notion further suggests that HCN activity would also not affect GluR in the same microvillus. In contrast, GluR activity would affect HCN channels as well as the GluR channels themselves in the same microvillus because Na^+^/K^+^ homeostasis around the GluR was not strictly regulated by the Na^+^/K^+^ pump (Figure 4B). Then, as demonstrated using Sodium Green Na^+^ imaging (Figure 6), the Na^+^ influx during *I*_GluR_ would transiently increase the Na^+^ concentration in the microvillus presumably because its volume is very small. However, K^+^ efflux through GluR channels during *I*_GluR_ would not cause any marked reduction in the K^+^ concentration in the microvillus because of the following reason. The microvilli with diameters of 0.2–0.5 μm and lengths of only 1.0–1.5 μm directly protruded from the cell bodies of MTN neurons (Figure 7) where the Na^+^/K^+^ concentrations remain unchanged, and thereby the K^+^ efflux through the GluRs with far smaller pore sizes compared to the neck diameter of the microvillus would be instantaneously and easily compensated for by the equivalent K^+^ influx from the soma. These assumptions were made for the simplification of the Na^+^ microdomain model (Figure 8A). Numerical calculations were performed using a two-compartment model in which an MTN neuron is composed of the soma and the microvillus compartments (Figure 8B).

In the present mathematical simulation, the reversal potential for *I*_GluR_ was variable because *I*_GluR_ was expressed by a Goldman-Hodgkin-Katz equation, and *E*_h_ was also variable depending on the concentrations of intracellular Na^+^ and K^+^ (see the Materials and Methods section). First, our model correctly simulated the effects of voltage-dependent modulation of *I*_h_ on *I*_GluR_. Similar to the real experiments illustrated in Figure 2, the amplitude of the *I*_GluR_ linearly increased with a negative shift of the holding potential when *I*_h_ was inactive [*I*_h_(−), Figure 8C], whereas the amplitude of the *I*_GluR_ increased with a negative shift of the holding potential to −80 or −90 and then turned to a decrease with further negative shifts of the holding potentials when *I*_h_ was active [*I*_h_(+), Figure 8C]. The voltage-dependent inhibition of *I*_GluR_ (Figures 8E–G) was also very similar to the real experiments (Figure 2E). *I*_GluR_ promptly decayed, whereas the baseline *I*_h_ reduction lasted longer than *I*_GluR_, which led to the generation of a transient outward current as reflected in the differential time-to-peaks of the *I*_GluR_ and *I*_h_ (Figure 8E). Under the voltage-clamp condition, the shunting effect no longer exists. Indeed, the *g*_Leak_ increase did not cause any decrease in the *I*_GluR_ (Figure 8H). However, the simultaneous increases in *g*_Leak_ and *I*_h_ (magenta curve) were less effective in suppressing the apparent *I*_GluR_ compared with the sole increase in *I*_h_ (red curve) (Figure 8H). This pattern was consistent with the comparison between the effect of 8-Br-cAMP (Figure 3D) and that of 8-Br-cGMP (see Supplementary Figure 2) which can activate TASK1 leak K^+^ current as well as *I*_h_ (Toyoda et al., 2010).

**Figure 9:**
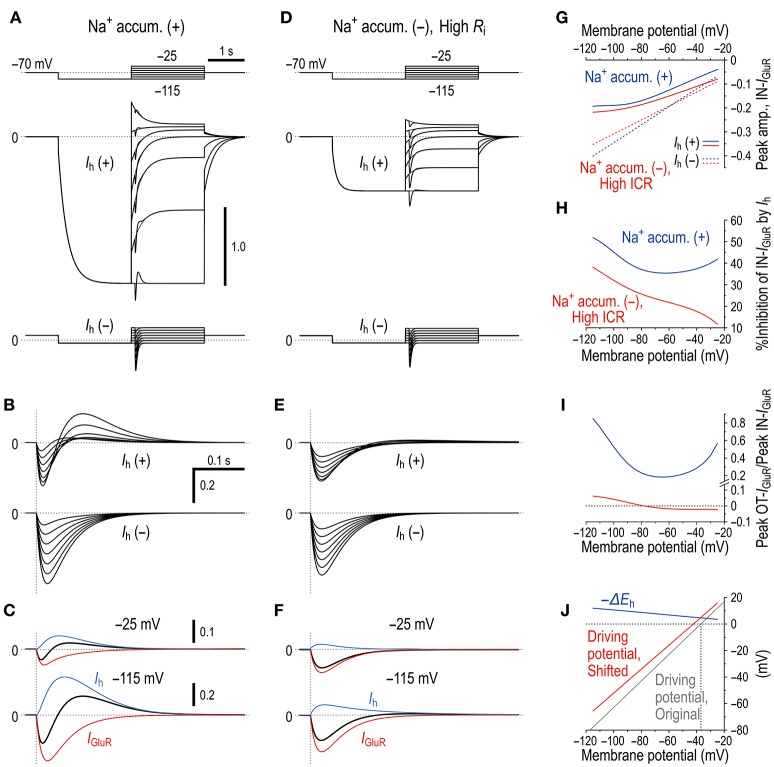
Microdomain model vs. *I*_h_ deactivation model. **(A)** In the Na^+^ microdomain model, *I*_GluR_ was evoked immediately after the membrane potential was positively stepped from −115 mV to varying membrane potentials but before *I*_h_ was largely deactivated in the presence and absence of *I*_h_ (upper and lower panels, respectively). The uppermost panel shows the voltage command pulses. **(B)** Superimposed traces of the *I*_GluR_ responses shown in **A**. **(C)** Separation of the pure *I*_GluR_ and *I*_h_ components of the apparent biphasic *I*_GluR_ composed of IN-*I*_GluR_ and OT-*I*_GluR_ obtained at −25 mV and −115 mV (upper and lower panels, respectively) as shown in **B**. **(D)** In the *I*_h_ deactivation model in which *R*_i_ was increased by 50 times from 5 × 10^5^ to 250 × 10^5^ (Ω) to create a space-clamp error. *I*_GluR_ was immediately after the membrane potential was positively stepped from −115 mV to varying potentials but before *I*_h_ was largely deactivated in the presence and absence of *I*_h_ (upper and lower panels, respectively). The uppermost panel shows the voltage command pulses. **(E)** Superimposed traces of the *I*_GluR_ responses shown in **D**. **(F)** Separation of the pure *I*_GluR_ and *I*_h_ components of the *I*_GluR_ composed of IN-*I*_GluR_ and a much smaller OT-*I*_GluR_ obtained at −25 mV and −115 mV (upper and lower panels, respectively) as shown in **B**. The biphasic profile of *I*_GluR_ is much less clear compared with that obtained in the Na^+^ microdomain model. **(G)** Relationships between the holding potentials and the peak amplitudes of IN-*I*_GluR_ obtained in the Na^+^ microdomain model and *I*_h_ deactivation model (blue and red curves, respectively) in the presence and absence of *I*_h_ (solid vs. interrupted curves, respectively). **(H)** A U-shaped profile of voltage-dependent inhibition of the peak IN-*I*_GluR_ in the microdomain model (solid blue line) vs. a linear monophasic profile of voltage-dependent inhibition of the peak IN-*I*_GluR_ in the *I*_h_ deactivation model (solid red line). **(I)** A U-shaped voltage-dependent change in the ratio of the peak amplitude of OT-*I*_GluR_ to that of IN-*I*_GluR_ with a minimal value of −65 mV in the Na^+^ microdomain model (blue curve) vs. a voltage-dependent non-linear monophasic change in the same ratio in the *I*_h_ deactivation model (red curve). Note the presence of negative ratios, which indicate that OT-*I*_GluR_ decreased to zero and then turned into a slow inward tail component of the preceding IN-*I*_GluR_. **(J)** A voltage-dependent maximum negative shift of *E*_h_ (black curve) caused by Na^+^ accumulation in the microvilli during *I*_GluR_ reduced the driving potential of *I*_h_ as revealed by a positive shift of the linear relationship between the membrane potential and the driving potential, which indicates that the generation of OT-*I*_GluR_ is due to either the decrease in the inward *I*_h_ or the increase in the outward *I*_h_. Compare the control (blue line) with that during *I*_GluR_ (red line).

Similar to the real experiment illustrated in Figure 5, *I*_GluR_ was smaller in amplitude and was followed by an outward current when *I*_GluR_ was evoked right after the holding potential was positively stepped from −115 mV to varying potentials but before *I*_h_ was largely deactivated, which contrasts with the *I*_GluR_ without *I*_h_ [compare *I*_h_(+) and *I*_h_(−) in Figures 9A,B,G]. This outward current was mediated either by a decrease in the baseline inward *I*_h_ or by an increase in the baseline outward *I*_h_ (Figure 9C), both of which are generated by negative shifts of *E*_h_ toward or away from the holding potentials (−115 and −25 mV; Figure 9J). Subsequently, a U-shaped voltage-dependent profile of the inhibition of IN-*I*_GluR_ can be observed (blue curve, Figure 9H). The ratio of the peak amplitude of OT-*I*_GluR_ to that of IN-*I*_GluR_ also displayed a prominent U-shaped voltage dependence with a minimum ratio at −65 mV (blue curve, Figure 9I), which is clearly indicative of a U-shaped voltage-dependent mechanism for the generation of OT-*I*_GluR_. Furthermore, the effects of space-clamp error were also simulated by introducing a large *R*_i_ between the soma and microvillus compartments (Figures 9D–F) because space-clamp error, which allows *I*_GluR_ to generate membrane depolarization, can consequently cause the deactivation of *I*_h_. In the *I*_h_ deactivation model, a large diminution of IN-*I*_GluR_ was accompanied only by a very small OT-*I*_GluR_ (Figures 9D–G). Furthermore, the *I*_h_ deactivation failed to simulate the U-shaped voltage-dependent inhibition of IN-*I*_GluR_ or the U-shaped voltage-dependent generation of OT-*I*_GluR_ (Figures 9H,I). Instead, the inhibition of IN-*I*_GluR_ decreased unidirectionally and nonlinearly with an increase in the membrane depolarization (red curve, Figures 9H), and the OT-*I*_GluR_ evoked at hyperpolarized membrane potentials turned out to be a slow inward tail component of the preceding IN-*I*_GluR_ at depolarized membrane potentials in the *I*_h_ deactivation model (red curve, Figure 9I). These observations clearly indicate that the present mechanism for the diminution of the IN-*I*_GluR_ and the U-shaped voltage-dependent generation of OT-*I*_GluR_ is distinct from the deactivation of *I*_h_ due to space-clamp error.

## Discussion

**Figure 10:**
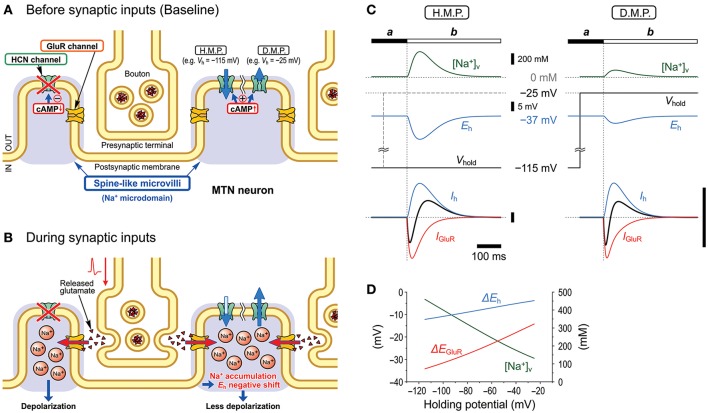
Schematic illustration showing the mechanisms underlying the inhibition of GluR responses by *I*_h_. Under the condition that *I*_h_ is activated/upregulated by membrane hyperpolarization or cAMP production, *I*_h_ changes in the right microvilli before **(A)** and during **(B)** synaptic inputs are illustrated. Na^+^ accumulation in the microvilli produced by Na^+^ influx through GluR channels negatively shifts *E*_h_ and consequently causes a decrease or increase in the driving potential of the inward or outward baseline *I*_h_, respectively, depending on the hyperpolarized (H.M.P.) or depolarized membrane potential (D.M.P.) levels, respectively **(C)**. Such changes in the baseline *I*_h_ curtail IN-*I*_GluR_ and generate the following OT-*I*_GluR_ **(C)**. The current calibration bars represent the same arbitrary unit. Peak [Na^+^]_V_ changes in the microvillus in response to the GluR activation at varying holding potentials. Changes of peak shifts of *E*_h_ and *E*_GluR_ following accumulation of Na^+^ in the microvillus at varying holding potentials **(D)**.

In the present study, by taking advantages of the morphological structure of MTN neurons that have round shaped somata from which short spine-like microvilli of 1.0–1.5 μm length directly protruded (Figure 7; also see Kang et al., 2004), whole-cell voltage-clamp recordings of GluR responses and *I*_h_ were obtained from MTN neurons with little space-clamp errors (Figures 2–5) while we also showed current-clamp recordings (Figure 1). Therefore, deactivation of *I*_h_ due to unclamped depolarization would not occur in MTN neurons under voltage-clamped conditions. Furthermore, the shunting of the membrane resistance due to conductance increases in HCN channels is incompatible with voltage clamp. *I*_GluR_ was markedly inhibited by the preceding activation of *I*_h_ in a U-shaped voltage-dependent manner with a minimal inhibition at approximately −60 mV (Figure 5), suggesting the existence of a mechanism distinct from the simple shunting effect or *I*_h_ deactivation mechanism. The U-shaped voltage-dependent generation of the outward current that curtails IN-*I*_GluR_ appeared to be mediated by a decrease in the baseline inward *I*_h_ at hyperpolarized membrane potentials and by an increase in the baseline outward *I*_h_ at depolarized membrane potentials (Figure 9C), which can be generated by negative shifts of *E*_h_ either toward or away from the holding potentials, respectively (Figure 10). The negative shift of *E*_h_ that varies depending on the holding potentials should be brought about by a transient accumulation of Na^+^ in the microvilli of MTN neurons in response to activation of GluRs (Figure 6). Furthermore, the mathematical modeling validated the negative shift of *E*_h_, and eliminated the possibilities of involvements of *I*_h_ deactivation or shunting in the inhibition of *I*_GluR_ (Figures 8–10).

### Transient accumulation of Na^+^ in the microvilli during *I*_GluR_ causes a negative shift of *E*_h_

In our previous study, we demonstrated that the Na^+^/K^+^ pump and HCN channels share a Na^+^ microdomain in spine-like microvilli and that there were bidirectional functional interactions between the Na^+^/K^+^ pump and HCN channels (Kang et al., 2004). The substitution of extracellular Na^+^ with Li^+^ increased *I*_h_ but almost abolished its tail current. This is because Li^+^ can flow through HCN channels into the microdomain but hardly or very slowly activates the Na^+^/K^+^ pump (Hermans et al., 1997; Féraille and Doucet, 2001), which consequently increases *I*_h_ but abolishes its tail-I due to the accumulation of Li^+^ that negatively shifts the *E*_h_. In contrast, ouabain enhanced not only *I*_h_ but also tail-I when examined at −90 mV (Figure 5 in Kang et al., 2004). Enhancements of *I*_h_ and its tail-I by ouabain indicate that not only the *I*_h_ but also its tail-I was being opposed or contaminated by the outward current mediated by Na^+^/K^+^ pump. Then, the possible decrease in the tail-I by a negative shift of *E*_h_ as a consequence of Na^+^ accumulation in microvilli by ouabain would be masked by the blockade of Na^+^/K^+^ pump outward current by ouabain. This may be the reason why the amplitude of tail-I evoked at −70 mV remained almost unchanged in spite of increase in *I*_h_ after application of ouabain (Figure 4A). Thus, depending on the balance between the degree of negative shift of *E*_h_ due to the accumulation of Na^+^ in the microvilli and the degree of inhibition of Na^+^/K^+^ pump current, the amplitude of tail-I would be changed following application of ouabain. Therefore, it is strongly suggested that Na^+^/K^+^ homeostasis around HCN channels is strictly regulated by the Na^+^/K^+^ pump, and the activity of HCN channels did not affect their reversal potential as long as the Na^+^/K^+^ pump was active in the same microdomain. However, this appears not to be the case with GluR because *I*_GluR_ was not enhanced but depressed by ouabain (Figure 4B). The present study demonstrated that GluR were co-localized with HCN channels in spine-like microvilli (Figure 7) and that GluR activation produced a transient accumulation of Na^+^ ions in the microvilli (Figure 6). Thus, when the Na^+^ influx through GluR is generated in addition to HCN activity, the Na^+^/K^+^ pump may not be able to afford to maintain Na^+^ concentration constant in the microvillus probably due to either the limited availability of the Na^+^/K^+^ pump or the differences in the Euclidean distance among the three channels that are colocalized in the same microvillus. Then, the *E*_h_ would be transiently shifted in the negative direction in response to GluR activation, which would lead to a reduction of the driving potential of the standing inward *I*_h_ at the resting or holding potential (< −70 mV). Because the standing inward *I*_h_ is reflected in the baseline current, the baseline current would shift outwardly during *I*_GluR_, and thereby cancel *I*_GluR_.

The diameter of spine neck in layer 2/3 pyramidal cells in the visual cortex ranged between 100 and 500 nm with a mean value of 200 nm (Arellano et al., 2007). Also in our study, spine-like microvilli neck diameters in electron microscopic observations in Figures 7C,D are about 200–250 and about 100 nm, respectively, although that appear larger in Figure 7E. The microvilli with lengths of 1.0–1.5 μm directly protruded from the cell bodies of MTN neurons. Regardless of the presence or absence of diffusion barrier, Na^+^ accumulation actually occurred in microvilli as demonstrated by Na^+^ imaging in the present study (Figure 6), although the diffusion barrier can modulate the time course of Na^+^ accumulation in microvilli. Furthermore, it is also known that stubby or mushroom type spines with large heads and thick necks can display larger responses to uncaged glutamate compared to thin spines which hardly display glutamate responses detectable in soma in spite of similar electrotonic distances (Matsuzaki et al., 2001). Given a transient accumulation of Na^+^ in dendritic spines, a similar modulation of *I*_GluR_ by HCN channels may occur in dendritic spines of cortical pyramidal neurons.

Non-synaptic GluRs may also exist in MTN neurons as glutamate responses have been found in the soma of spinal dorsal root ganglion neurons (Huettner, 1990) or trigeminal ganglion neurons (Sahara et al., 1997). If non-synaptic GluRs are expressed in extra-microvilli in MTN neurons, glutamate puff would also activate these GluRs. Then, this may preclude us from drawing the present conclusion. However, an electron microscopic study demonstrated the embracement of MTN neurons by astrocytic processes that only allow synaptic contacts on the neuronal surface and protect MTN neurons from non-synaptic input (Copray et al., 1990a). This is in contrast to the somatic synapses on most brainstem motoneurons and interneurons lacking any astrocytic wrapping (Copray et al., 1990a), which would allow ambient GABA or glutamate to activate extra-synaptic receptors, causing tonic currents. This would not happen in MTN neurons due to the embracement by astrocytic processes, and therefore extra-synaptic receptors would not exist in MTN neurons. Nevertheless, a further study to selectively activate synaptic GluRs may be necessary to draw a definitive conclusion.

### Na^+^ accumulation in response to GluR activation by puff application vs. physiological activity of presynaptic terminals

Puff application of glutamate or AMPA may activate most of GluRs expressed in a single MTN neuron, the number of which may be much larger than that of GluRs activated in response to glutamate release from presynaptic terminals of a certain specific input. Subsequently, the total Na^+^ influx from GluRs in response to puff application may be much larger than that caused by physiological activity of glutamatergic inputs. Given a negative shift of *E*_h_ due to a large accumulation of Na^+^ by puff application, a question arises whether physiological activity of glutamatergic inputs can cause a similar negative shift of *E*_h_ because the accumulation of Na^+^ might be much smaller than that caused by puff application of glutamate or AMPA. Especially, a large accumulation of Na^+^ as a result of many GluRs activation by puff application may lead to a saturation of Na^+^/K^+^ pump activity that facilitates Na^+^ accumulation, whereas such saturation would not occur under physiological activity.

However, Na^+^ accumulation in microvilli by GluR activation is not a result of saturation of Na^+^/K^+^ pump activity, as revealed by the differential effects of ouabain on *I*_GluR_ and *I*_h_ (Figure 4), and by Na^+^ imaging (Figure 6). The soma of MTN neurons is covered by numerous microvilli (Liem et al., 1991; Lazarov, 2002), and synaptic GluRs are sparsely distributed in respective microvilli (Figure 7; Paik et al., 2012). Activation of these GluRs by bath application of glutamate at 1 mM caused increases in Na^+^ concentration only beneath the cytoplasmic membrane in the microvilli distributed over the soma, leaving the Na^+^ concentration in the cytoplasm almost unchanged (Figure 6). Saturation of Na^+^/K^+^ pump activity occurred after 3 min bath application of glutamate as revealed by the increase in Na^+^ concentration not only in the microvilli but also partly in the cytoplasm close to cytoplasmic membrane, but hardly occurred in response to 30-s bath application (Figure 6E). Therefore, saturation of Na^+^/K^+^ pump activity would not occur in response to 50 or 200 ms puff application of glutamate or AMPA at 5–20 times smaller concentration (50–200 μM) than that of bath application.

Furthermore, ouabain differentially affected *I*_h_ and *I*_GluR_ (Figure 4): Enhancement of *I*_h_ by ouabain indicates that Na^+^/K^+^ pump was activated immediately by the influx of Na^+^ through HCN channels. In contrast, inhibition of *I*_GluR_ by ouabain indicates that Na^+^ influx through GluRs did not apparently stimulate Na^+^/K^+^ pump. *I*_GluR_ is either decreased due to an increase in the basal level of Na^+^ concentration caused by inhibition of Na^+^/K^+^ pump by ouabain or opposed by an enhancement of *I*_h_ as a result of the inhibition of Na^+^/K^+^ pump by ouabain, in a manner similar to the case with 8-Br-cAMP. Because the OT-*I*_GluR_ was also enhanced by ouabain, the inhibition of IN-*I*_GluR_ by ouabain was at least partly due to the generation of outward current mediated by a transient reduction of the enhanced (inwardly shifted) baseline *I*_h_ by ouabain during *I*_GluR_ that might have led to the generation of OT-*I*_GluR_. This strongly suggests that the activity of Na^+^/K^+^ pump is not saturated yet even during or at the offset of *I*_GluR_. Therefore, it is likely that the accumulation of Na^+^ during *I*_GluR_ is not caused by the saturation of Na^+^/K^+^ pump activity.

The observed inhibition of *I*_GluR_ by *I*_h_ is a whole-cell current generated as a result of summation of the small current changes independently occurring in the respective microvilli. Even if most of GluRs in an MTN neuron are activated by puff application of glutamate or AMPA, Na^+^ concentrations in the respective microvilli would not increase proportionally with the total number of activated GluRs in an MTN neuron, because respective GluRs in respective microvilli do not contribute to the accumulation of Na^+^ in the cytoplasm, but separately and independently causing Na^+^ increase in the respective microvilli. Thus, the total number of activated GluRs is not reflected in the concentration of Na^+^ in the cytoplasm or respective microvilli. Repetitive stimulation which can be mimicked by 50- or 200-ms puff application is more effective in inducing *I*_GluR_ inhibition by HCN channels compared to single stimulation of GluR (Carr et al., 2007), suggesting that HCN2, rather than or in addition to HCN1, may be involved in this inhibition.

### Deactivation of *I*_h_ is not compatible with the U-shaped voltage-dependent inhibition of EPSCs

The outward current that follows the inward glutamatergic current is very similar to the hyperpolarization that follows EPSPs in many cell types, and the hyperpolarization was considered to be caused by deactivation of *I*_h_ due to EPSPs (Magee, 1998, 1999; Santello and Nevian, 2015). However, this idea is not necessarily correct but yet to be addressed. It is certain that the peak amplitudes of EPSPs would be decreased due to the deactivation of HCN channels during the rising phase of EPSPs if EPSPs are evoked from a potential where HCN channels are active. However, EPSPs would not be followed by HCN-mediated afterhyperpolarizations. This is because during the decay phase of EPSPs, the deactivation of *I*_h_ would be replaced with the voltage-dependent activation of *I*_h_. The apparent sensitivity of the hyperpolarization following EPSPs to the HCN blocker ZD7288 may be simply due to the ZD7288-induced hyperpolarization of the baseline membrane potential, which consequently decreases the membrane hyperpolarization even if K^+^ channels are responsible for the hyperpolarization. Thus, the underlying mechanism is not clear in cortical pyramidal cells.

There may be a possibility that the transient outward current following the glutamate puffs at negative voltages is artificially caused by a space-clamp error that allowed a transient deactivation of *I*_h_ due to a possible unclamped membrane depolarization evoked by *I*_GluR_. However, this possibility is very small because the space-clamp error can be considered to be negligible due to a very short electrotonic length of the microvilli of 1.0–1.5 μm in length and 0.2–0.5 μm in diameter that protrude directly from the soma (Figures 7B,C,E). More importantly, the transient outward current following the glutamate puffs displayed a U-shaped voltage dependence (Figures 5E,F). Because the baseline *I*_h_ is likely to be very close to zero or an outward current at such depolarized membrane potentials when the reversal potential for *I*_h_ is −27 or −37 mV, its deactivation due to the possible membrane depolarization by *I*_GluR_ would have resulted in the generation of either no outward current or an apparent inward current, contrary to what was observed in the present study (Figures 5E,F). Indeed, the mathematical modeling of the *I*_h_ deactivation as was the case with pyramidal cells revealed that despite a large diminution of IN-*I*_GluR_ only a negligibly small outward current could have been caused by the deactivation of *I*_h_ brought about by creating a large space-clamp error (Figures 9E,F), and neither the diminution of IN-*I*_GluR_ nor the generation of OT-*I*_GluR_ displayed a U-shaped voltage-dependent nature (Figures 9H,I).

### Possible shunting effects on *I*_GluR_ under voltage-clamp conditions

Under the current-clamp condition, the amplitudes of the EPSPs would be decreased by decreasing the input resistance. However, under the voltage-clamp conditions, EPSCs would remain constant despite changes in input resistance unless there was charge redistribution that can be seen in cortical pyramidal cells during the activation of synaptic inputs onto the spines of the apical dendrites due to the space-clamp problem. Because in MTN neurons spine-like microvilli directly protrude from the soma, space clamp in spine-like microvilli is much more stringent than that in dendritic spines that protrude from the apical dendrites of cortical pyramidal cells. Nevertheless, *I*_GluR_ was reduced by the activation of HCN channels in MTN neurons. In the present study, there were three lines of evidence against the possible involvement of the shunting effects of *I*_h_. First, shunting effects would not cause outward currents following *I*_GluR_ (Figures 2–5). Second, 8-Br-cAMP application suppressed the GluR-activated bursting without affecting the current threshold for evoking spikes by current pulse injections (Figure 1). Third, the mathematical modeling of the simultaneous activation of *I*_h_ and leak K^+^ current, which certainly has shunting effects, revealed decreases in the inhibitory effect of *I*_h_ on *I*_GluR_ (Figure 8H), which was consistent with the results of the experiment (Supplementary Figure 2). Contrary to the present findings, it has been reported that activation of NO-cGMP signaling pathway enhanced NMDA current through gating HCN channels in CA1 hippocampal pyramidal neurons (Neitz et al., 2014).

### Functional significance of *I*_GluR_ inhibition by *I*_h_ in MTN neurons

In MTN neurons, *I*_h_ activation hampered glutamatergic synaptic impacts and thereby suppressed glutamate-induced burst firing (Figure 1). Activation of serotonin receptors in MTN neurons has been reported to cause cAMP production and inhibit persistent *I*_Na_ (*I*_NaP_) that mediates bursting in MTN neurons (Tanaka and Chandler, 2006). Therefore, such synaptic action would also enhance *I*_h_ to inhibit *I*_GluR_ and prevent MTN neurons from *I*_NaP_-mediated bursting. Subsequently, MTN neurons would be kept in primary sensory neuron mode, which faithfully conveys proprioceptive information to the central nervous system. This notion is consistent with the previously proposed mechanism (Saito et al., 2006; Kang et al., 2007) for voltage-dependent switching of the functional modes of MTN neurons between the primary sensory neuron single spiking and the premotor neuron bursting modes through the voltage-dependent activities of 4-aminopyridine-sensitive A-type K^+^ currents in the soma and riluzole-sensitive low-threshold *I*_NaP_ in the stem axon.

Recently, we have reported that protein kinase C activation by metabotropic glutamate receptors enhanced burst firing through the enhancement of resonance by upregulating *I*_NaP_ in MTN neurons (Chung et al., 2015). Taken together, it is suggested that the activities of *I*_h_, 4-aminopyridine-sensitive A-type K^+^ currents, and *I*_NaP_ cooperatively contribute to switching between the two modes. It is of interest to investigate whether there are any synaptic inputs to inhibit HCN in MTN neurons. It is already known that terminals arising from the nucleus locus coeruleus exert noradrenergic synaptic action on MTN neurons (Copray et al., 1990b; Takahashi et al., 2010). These noradrenergic inputs may inhibit HCN activity by downregulating cAMP production through the activation of α2A adrenergic receptors (Wang et al., 2007) and consequently facilitate burst firing in response to glutamatergic inputs in MTN neurons, which is implicated in the attack behavior by biting enemies (Copray et al., 1990b; Takahashi et al., 2010). These functions are crucially mediated by Na^+^ microdomain in which the Na^+^/K^+^ pump, HCN and GluR functionally interact one another. This novel mechanism highlights a possible involvement of an impaired functional coupling between HCN channels and the Na^+^/K^+^ pump in a variety of neurological disorders also in other brain regions.

## Author contributions

YoK: Conceived and designed the research; MS, YaK, JW, HS, and HT: Conducted the electrophysiological experiments; JB, EK, TK, and YB: Conducted the immunohistochemical experiments; TT, MS, and YoK: Performed the numerical simulation study; All authors analyzed the data; MS, YaK, MK, SO, YB, and YoK: Wrote the manuscript. All authors have given approval to the final version of the manuscript.

### Conflict of interest statement

The authors declare that the research was conducted in the absence of any commercial or financial relationships that could be construed as a potential conflict of interest.

## Abbreviations

8-Br-cAMP: 8-bromoadenosine 3′,5′-cyclic monophosphate sodium salt
8-Br-cGMP: 8-bromoguanosine 3′,5′-cyclic monophosphate sodium salt
*E*_h_: reversal potential for *I*_h_
*E*_K_: reversal potential for K^+^ currents
*G*_h_: conductance of HCN channels
GluR: glutamate receptor
HCN: hyperpolarization-activated cyclic nucleotide-gated
*I*_GluR_: GluR-mediated currents
*I*_h_: current mediated by HCN channels
*I*_NaP_: persistent Na^+^ current
IN-*I*_GluR_: inward component of the *I*_GluR_
IQR: interquartile range
MTN: mesencephalic trigeminal nucleus
N-ACSF: normal ACSF
OT-*I*_GluR_: outward component of the *I*_GluR_
PB: phosphate buffer
PND: postnatal day
VGLUT: vesicular glutamate transporter.
