# Supplementary material for: Inhibition of GluR Current in Microvilli of Sensory Neurons via Na+-Microdomain Coupling Among GluR, HCN Channel, and Na+/K+ Pump
Source: Front Cell Neurosci. 2018 Apr 24;12:113. doi: 10.3389/fncel.2018.00113 (PMC5928758; doi:10.3389/fncel.2018.00113)
Supplement: Supplementary file 1 [file Data_Sheet_1.pdf]

Supplementary Material

Inhibition of GluR Current in Microvilli of Sensory Neurons via Na^+-Microdomain Coupling among GluR, HCN channel and Na^+/K^+ Pump

Yasuhiro Kawasaki, Mitsuru Saito*, Jonghwa Won, Jin Young Bae, Hajime Sato, Hiroki Toyoda, Eriko Kuramoto, Mikihiko Kogo, Takuma Tanaka, Takeshi Kaneko, Seog Bae Oh*, Yong Chul Bae and Youngnam Kang*

*** Co-correspondence:** Mitsuru Saito, mtrsaito@dent.kagoshima-u.ac.jp; Seog Bae Oh, odolbae@snu.ac.kr; Youngnam Kang, kang@dent.osaka-u.ac.jp

Supplementary Figures

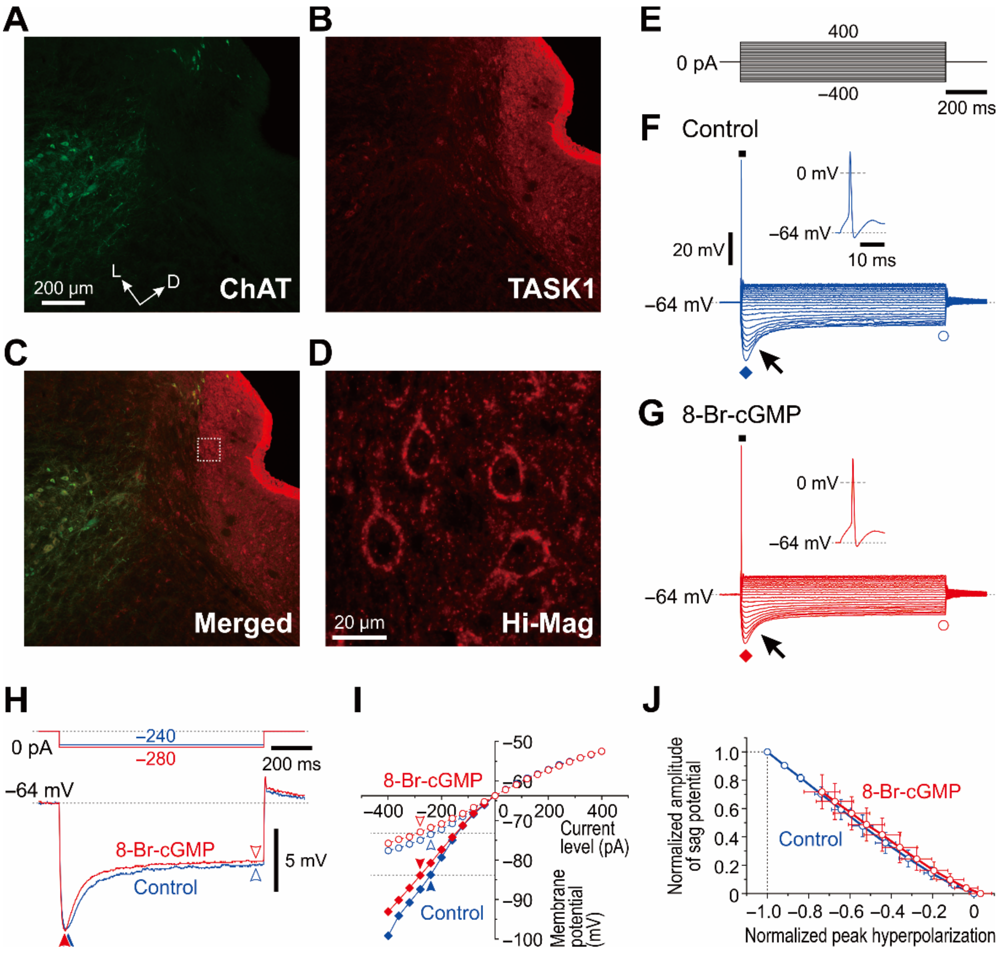

Supplementary Figure 1 | Expression of TASK channels that act as leak K^+ channels and the effects of 8-Br-cGMP that activates HCN and TASK channels on the input resistance. **(A–D)** Immunoreactivities for choline acetyltransferase (ChAT) (*A*) and TASK1 **(B)** in the trigeminal motor nucleus (TMN) and in the MTN. Merged image **(C)**. The area enclosed by a rectangle in **(C)** is enlarged in **(D)**. Note the expression of TASK channels in MTN neurons as well as in TMN neurons. **(E)** Injected current pulses at the resting membrane potential of −64 mV. **(F)** and **(G)**, Membrane potential responses to current pulse injections **(E)** before **(F)** and after the bath application of 8-Br-cGMP **(G)**. *Insets*, Enlarged traces of action potentials seen during the respective time periods indicated with short horizontal bars in **(F)** and **(G)**. Note the prominent I_h-mediated sag potentials in the MTN neurons. **(H)** Superimposed traces of the responses obtained before (blue traces) and after 8-Br-cGMP application (red traces), the negative peaks of which were almost the same (filled arrowheads), while a slight enhancement of the sag potential was observed after 8-Br-cGMP application (open arrowheads). Note that the sag potential became slightly more prominent, indicating that I_h was slightly enhanced by 8-Br-cGMP application. **(I)** *I-V* relationships measured at the timing of the negative peaks of the sag potentials (filled diamonds) and at the end of the current pulses (open circles) obtained before (blue) and after 8-Br-cGMP application (red). Note that 8-Br-cGMP significantly ($P < 0.001$, $n = 7$) decreased the input resistance (from 100 ± 24 to 74 ± 26 MΩ; compare blue and red filled diamonds). **(J)** Relationships between the normalized amplitude of the negative peaks and that of the sag potentials in response to the respective negative current pulses obtained before (blue circles) and after 8-Br-cGMP application (red circles) (n = 7; mean ± SD). The normalized

maximum hyperpolarization and maximum amplitude of the sag potentials were reduced by 27% ± 9% ($P < 0.001$) and 28% ± 12% ($P < 0.002$), respectively. An insignificant slight shift of the relationship in the right direction from −66.7 ± 2.5 to −65.0 ± 4.0 mV ($P > 0.2$) occurred despite the significant reduction of the input resistance following 8-Br-cGMP application **(I)**.

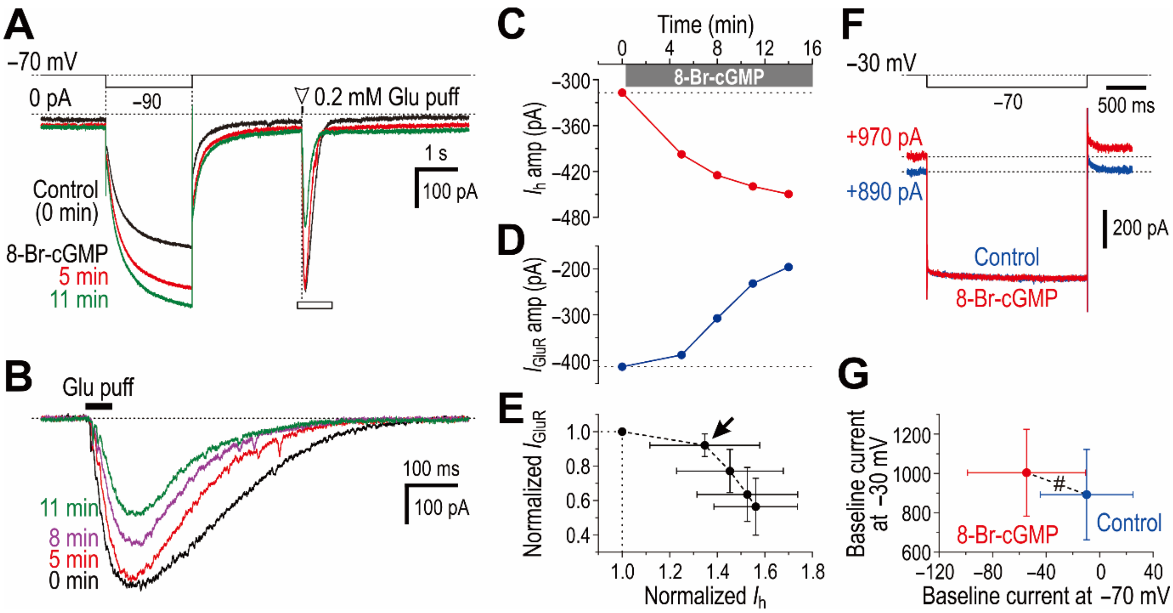

Supplementary Figure 2 | Effects of the activation of leak K^+ currents and I_h by 8-Br-cGMP on I_{GluR}. **(A)** and **(B)** Current responses to a negative voltage pulse (−20 mV, 500 ms) and the subsequent 50-ms puff application of 0.2 mM glutamate recorded before (black trace) and 5 min (red trace) and 11 min (green trace) after the application of 8-Br-cGMP under the voltage-clamp condition at −70 mV **(A)**. Enlarged traces **(B)** of current responses to the glutamate puff shown in **(A)**. The baseline current levels were aligned **(B)**. **(C)** and **(D)** Plot of the amplitudes of I_h and I_{GluR} against time. Note that the bath application of 8-Br-cGMP decreased the amplitude of I_{GluR} and concomitantly increased the amplitude of the apparent I_h. **(E)** Plot of the normalized amplitudes of I_{GluR} (black filled circles) against those of I_h obtained following the application of 8-Br-cGMP (200 μM; $n = 5$; mean ± SD). Note that the amplitude of I_{GluR} decreased non-linearly with increases in the amplitude of I_h, which was similar to the case with 8-Br-cAMP (**Figure 3D**). The bath application of 8-Br-cGMP significantly increased I_h by 56% ± 18% ($P < 0.02$) and decreased I_{GluR} in amplitude by 44% ± 17% ($P < 0.02$). When the normalized I_h was increased to 1.21 ± 0.08 by 8-Br-cAMP, the normalized I_{GluR} was decreased to 0.86 ± 0.03 (**Figure 3A–F**), which was more prominent than the change (arrow) caused by 8-Br-cGMP (normalized I_h = 1.35 ± 0.23, the normalized I_{GluR} = 0.92 ± 0.07). **(F)** Current responses to negative voltage pulses (−40 mV, 2 s) obtained before (blue traces) and after the application of 8-Br-cGMP (red traces) under voltage-clamp conditions at −30 mV where I_h is completely deactivated. The baseline current shifted outward, and the leak current increased. **(G)** Relationship between the mean (± SD) baseline current level at −70 mV and that at −30 mV obtained before (blue circle) and after 8-Br-cGMP application (red circle). *: $P < 0.02$. 8-Br-cGMP shifted the baseline current at −30 mV outward by 112 ± 59 pA ($P < 0.02$), but at −70 mV inwardly by 45 ± 20 pA ($P < 0.02$), indicating the simultaneous activation of leak K^+ current and I_h. Wilks' lambda test also revealed a significant difference (#, $P < 0.003$) in the distribution of the baseline currents at −30 and −70 mV in the two-dimensional space between the responses obtained before and after 8-Br-cGMP application.
